# Anxiety among secondary school students in the war-torn Tigray, Ethiopia, 2024: A call for action

**DOI:** 10.1371/journal.pmen.0000526

**Published:** 2026-03-18

**Authors:** Haftom Tesfay Gebremedhin, Abadi Kidanemariam Berhe, Yemane Gebremariam Gebre, Alem Gebremariam, Mulu Ftwi Beraki, Tesfay Gebreslassie Gebrehiwot, Guesh Teklu Woldemariam, Embay Amare Alemseged, Haileslassie Tesfay Tadese, Yemane Berhane Tesfau

**Affiliations:** 1 Department of Psychiatry, College of Medicine and Health Sciences, Adigrat University, Adigrat, Tigray, Ethiopia; 2 Department of Public Health, College of Medicine and Health Sciences, Adigrat University, Adigrat, Tigray, Ethiopia; 3 Tigray Health Research Institute, Mekelle, Tigray, Ethiopia; 4 Department of Pediatrics, College of Medicine and Health Sciences, Adigrat University, Adigrat, Tigray, Ethiopia; 5 Department of Midwifery, College of Medicine and Health Sciences, Adigrat University, Adigrat, Tigray, Ethiopia; 6 Department of Nursing, College of Medicine and Health Sciences, Adigrat University, Adigrat, Tigray, Ethiopia; FSBSI Scientific Research Institute of Neurosciences and Medicine: FGBNU Naucno-issledovatel'skij institut nejronauk i mediciny, RUSSIAN FEDERATION

## Abstract

Adolescents are especially vulnerable to stress and trauma. Exposure to armed conflict significantly raises the risk of anxiety, which often lasts into the post-conflict phase. The war in northern Ethiopia has caused widespread trauma, displacement, and destruction of services. Understanding mental health after conflict is crucial for guiding recovery efforts, informing school-based programs, and shaping local public health priorities. However, data on the prevalence of post-war anxiety among secondary school students in the region is lacking. This study assessed the prevalence and factors associated with clinically significant anxiety among secondary school students in Tigray, Ethiopia. A school-based cross-sectional study was conducted among 608 randomly selected secondary school students in Adigrat Town, Tigray, Ethiopia. Data was collected using a structured self-administered questionnaire, including the Generalized Anxiety Disorder (GAD)-7 scale for anxiety assessment. Multivariable binary logistic regression was used to identify factors associated with clinically significant anxiety symptoms. The prevalence of clinically significant anxiety symptoms among students was 32.7% (95% CI: 28.9%, 36.5%). Among sociodemographic factors, being female (AOR = 6.52, 95% CI: 3.70, 11.46) and age ≥ 18 years (AOR = 3.19, 95% CI: 1.60, 6.39) were significantly associated with anxiety symptoms. Trauma-related experiences, including combat or exposure to a battlefield (AOR = 5.05, 95% CI: 1.95, 13.12), physical violence (AOR = 2.25, 95% CI: 1.32, 3.82), bullying (AOR = 2.26, 95% CI: 1.24, 4.11), and contact sexual abuse (AOR = 3.97, 95% CI: 1.58, 9.96), were significantly associated with anxiety symptoms. Suicidal ideation (AOR = 4.01, 95% CI: 1.96, 8.24) and depression (AOR = 3.99, 95% CI: 2.32, 6.87) were also significantly associated with anxiety symptoms. This study reveals a high prevalence of clinically significant anxiety symptoms among secondary school students, associated with gender, age, trauma exposure, and comorbid conditions. These findings highlight the need for school-based mental health screening and trauma-informed support to reduce long-term psychological effects in this vulnerable generation.

## Introduction

Armed conflict is a serious public issue, with more than a billion young people living in affected areas, predominantly in lower- and middle-income countries where 90% of the world’s children and adolescents reside [1,2]. One in four young people globally is affected by armed conflict or disaster, often with little to no access to psychological support [3]. During post-conflict or war, an estimated one in five people has mental problems [4]. Children and youths exposed to conflict face a heightened risk of developing mental health problems, especially anxiety, depression, and post-traumatic stress disorder (PTSD) [5]. Anxiety is the hallmark of anxiety disorders and is an unmanaged, pervasive, unpleasant, and enduring state of negative emotion, marked by apprehensive anticipation of unforeseen and unavoidable future threats, accompanied by physical tension and heightened alertness [6]. Anxiety disorders are among the most common and impairing conditions in adolescence, often emerging early in life and predicting poorer educational performance, reduced general functioning, and long-term mental health difficulties [7–9].

Multiple factors contribute to the development of anxiety. Individual traits and exposure to stressful life events increase susceptibility to anxiety [10,11]. Trauma exposure, in particular, can lead to long-term mental health issues by causing physiological changes that create individual vulnerabilities to various mental health problems later in life [12]. As a result, prolonged exposure to stressors associated with conflict not only causes anxiety immediately but also significantly increases the risk of developing later PTSD [13]. Evidence indicates that adverse experiences can become biologically embedded; for instance, altered amygdala connectivity in children exposed to stress predicts future anxiety symptoms [14,15]. Early life stress is also associated with hyperactive hypothalamic-pituitary-adrenal (HPA) axis responses, which are connected to later anxiety symptoms [16,17]. This is supported by neurobiological research indicating that trauma-related changes in this system increase stress reactivity and cause ongoing anxiety [18].

Studies from populations affected by conflict indicate that anxiety symptoms frequently worsen over time. A prospective study of children and adolescents exposed to war found that anxiety symptoms increased over time. [19]. Similarly, among Syrian and Iraqi refugee youth resettled in the US, anxiety prevalence was 38% upon arrival and worsened over two years [20]. Globally, the prevalence of anxiety among adolescents during or after violence varies widely, from 23.7% to 94.9% [21–24]. In Ethiopia, a post-war study among high school students revealed a prevalence of 39.7%, highlighting the possible burden of mental illness in areas affected by conflict [25].

In non-conflict settings of Ethiopia, adolescent anxiety levels are much lower. A systematic review among children and youth showed prevalences ranging from 0.5% to 23% [26], and an Ethiopian school-based study in a stable area found a prevalence of 25.05% among secondary school students [27]. Even in Tigray, the prevalence of anxiety before the conflict was 11.48%, showing typical levels before the war started. However, during the conflict, a phone survey found that anxiety prevalence increased to 34.43% [28]. This represents a threefold increase and highlights the severe psychological effects of the war on young people.

The Tigray region in northern Ethiopia faced severe and ongoing suffering during the two-year siege and war. The population endured profound trauma following the war [29], including the forced displacement of over 2 million people [30], the deliberate destruction of 70–80% of health facilities and targeted attacks on health workers [31,32], widespread gender-based violence [33], and a prolonged, complete blockade of communication, electricity, and other necessities [29]. These conditions create severe ecological and psychosocial stress that can increase the risk of anxiety among young people.

The development of anxiety is affected by different demographic, behavioral, and trauma-related factors. Studies have shown that a higher prevalence of anxiety was observed among females and individuals with lower socioeconomic status [34,35]. Moreover, studies reported that being female [22,25,36,37], age [22,38,39], being in a higher grade in school [40], smoking [41], alcohol use [27,42], witnessing the murder of family/friends [25], and depression [25,27,43] have a positive relationship with anxiety. Furthermore, individuals with suicidal ideation exhibit higher anxiety symptom severity compared to those without it [44]. Understanding these factors in post-conflict settings is crucial for creating effective prevention and intervention strategies.

To understand these complex relationships, this study relies on Bronfenbrenner’s Ecological Systems Theory [45] and the Transactional Model of Stress and Coping [46]. According to Bronfenbrenner’s framework, war affects an adolescent’s surroundings on several levels, ranging from community disruption in the exosystem to direct trauma in the microsystem [45]. According to the Transactional Model, people’s mental health outcomes are shaped by how they cognitively evaluate and manage stressors [46,47]. When taken as a whole, these theories clarify how ecological disruptions caused by war interact with individual vulnerabilities and coping mechanisms to affect the trajectories of post-conflict anxiety [47–50].

A high prevalence of anxiety among young people was identified in a phone survey conducted during the ongoing war. Still, no school-based assessment has been performed since the conflict ended. Little is known about the extent of anxiety and related factors among adolescents after the Pretoria Peace Agreement, despite the challenging humanitarian situation in Tigray. Therefore, essential questions remain: What is the current level of anxiety among secondary school students in Tigray after the war, and what factors are significantly associated with it?

This pre-registered cross-sectional study, conducted in 2024, aims to fill this gap by assessing the prevalence of clinically significant anxiety symptoms and its associated factors among secondary school students in the Tigray Region. By comparing data from before and during wartime, this research offers valuable insights for policymakers, mental health professionals, and school systems seeking to support adolescent recovery in one of the world’s most severely affected conflict zones.

## Methods and materials

### Study design, setting, and period

A school-based cross-sectional study was conducted from November 1–30, 2024, in Adigrat Town, one of the zonal capitals of the Tigray Region in northern Ethiopia. The town has four public and one private secondary school.

### Study participants

All 10,714 students from public and private secondary schools (grades 9–12) enrolled in the 2024–2025 academic year, comprising 4,671 males and 6,043 females, were included in the study population. Public schools accounted for 8,914 of these students. The age range of participants extended up to 24 years. This shows the disruption to the regional education system caused by the war, which led to delayed school progression for many students.

This study included students who had lived in the region before the Pretoria Agreement (a permanent ceasefire) and were attending classes during data collection. Students who were seriously sick during the data collection were excluded. For this study, ‘seriously sick’ was operationally defined as a student who was visibly unable to provide meaningful assent or complete the questionnaire due to an acute condition like a high fever, vomiting, severe pain, or an active psychotic episode.

### Sample size and sampling technique

The sample size was calculated for the target population of secondary school students in Adigrat Town using the single-proportion formula. The calculation was based on the following assumptions: an anxiety proportion of 39.7%, taken from a study of conflict-affected school adolescents in Woldia Town [25]; a 95% confidence interval; a 5% margin of error; a 10% non-response rate; and a design effect of 1.5. Accordingly, the total sample size for this study was 608 study**.**

A stratified simple random sampling design with proportional allocation was employed. First, the schools were stratified into public and private secondary schools, and the sample size was proportionally allocated based on each school’s student enrollment. Within each school type, students were further stratified by academic grade into 9th, 10th, 11th, and 12th grades, and proportional allocation was again used to ensure that the probability of selection remained equal across all strata.

Each stratum used complete and up-to-date student rosters with distinct identification numbers as the sampling frame. Using a computer-generated series of random numbers applied to the student identification numbers, the predefined number of participants for each grade-level stratum was chosen from these complete lists. Students were selected individually rather than in classroom clusters, and no volunteers were requested. The randomly selected participants were then invited to a comfortable setting, and they completed the self-administered questionnaires after an orientation.

### Data collection and instruments

Four health professionals with master’s degrees were adequately oriented to study participants to complete the structured, self-administered questionnaire, which comprised four sections. The first section assessed participants’ sociodemographic characteristics. The second part of the questionnaire was the General Anxiety Disorder-7 (GAD-7). The use of this tool is well supported by its psychometric validation in Ethiopia. The GAD-7 has shown strong internal consistency (Cronbach’s α = 0.77, McDonald’s Omega = 0.77, 0.78). It has a unidimensional factor structure confirmed by factor analyses (CFI = 1.000, GFI = 1.000, RMSEA = 0.037) and established convergent and divergent validity. Additionally, it has shown consistent item analysis and measurement invariance across gender. This confirms its suitability for use with young populations in Ethiopia [51]. The tool has seven items, and each item is rated on a four-point Likert scale that ranges from 0, “not at all,” to 3, “nearly every day.” The scores of all items were summed to yield a total score ranging from 0 to 21. Based on the total score, anxiety symptom severity was categorized as minimal (0–4), mild [5–9], moderate [10–14], and severe [15–21,52,53]. In accordance with the standard international and Ethiopian validation cut-offs, a GAD-7 score of 10 or higher indicates the presence of clinically significant anxiety symptoms, while scores below 10 indicate no anxiety [51,52]. The current study found a Cronbach’s alpha coefficient of 0.917, indicating excellent internal consistency.

The third part of the questionnaire assessed behavioral factors. Substance use, such as khat, tobacco, and alcohol, was evaluated using a yes-or-no question adapted from the Alcohol, Smoking, and Substance Involvement Screening Test (ASSIST) tool [54,55], a globally validated instrument with strong psychometric properties. It showed good internal consistency with Cronbach’s α values ranging from 0.77 to 0.94 across substance categories and 0.89 for the Total Substance Involvement score [56]. Physical activity was assessed by a single question to determine whether the participants met the WHO-recommended level or not [57].

The final segment of the questionnaire was regarding psychosocial factors, which included sexual abuse, suicidality, bullying experiences, trauma exposure, depression, and social support. Lifetime exposure to sexual abuse was measured with four questions from the ISPCAN Child Abuse Screening Tool Children’s Version [58]. Suicidal ideation and attempts were assessed using questions adapted from the World Mental Health Composite International Diagnostic Interview (WMH-CIDI) [59]. The adapted questions had “Yes/No” answers and were self-administered. Bullying experiences were assessed by a single-item measure adapted from the WHO/CDC Global School-based Health Survey (GSHS) [60]. Trauma exposure histories were assessed using a tool question adapted from the PCL-5 Life Events Checklist, designed to identify events meeting DSM-5 Criterion A [61]. Participants answered “Yes” or “No” to a list of event types. Depression symptoms were assessed using the 9-item Patient Health Questionnaire (PHQ-9), a validated scale scoring each item from 0 (“not at all”) to 3 (“nearly every day”), with total scores ranging from 0 to 27 [62]. A cut-off score of ≥10 indicated the presence of likely clinically significant depressive symptoms [63]. In our sample, the PHQ-9 demonstrated good reliability (Cronbach’s α = 0.88). Social support was assessed using the Oslo-3 Social Support Scale (OSS-3), yielding a sum score ranging from 3 to 14 [64]. The total score from the OSSS-3 was categorized as follows: 3–8 indicated poor social support, 9–11 indicated moderate social support, and 12–14 indicated strong social support [65].

### Operational definition

**Anxiety:** Participants who scored 10 or higher out of 21 on the GAD-7 were considered to have anxiety, while those who scored below 10 were classified as not having clinically significant anxiety symptoms (moderate to severe) [52].
**Depression:** Participants who scored ≥10 out of 27 on the PHQ-9 were classified as having clinically significant depressive symptoms (moderate to severe) [63].
**Bullying**: Being bullied refers to a student’s exposure to repeated incidents of bullying in the last 30 days. Bullying is defined as intentional and repeated aggression, including verbal (e.g., teasing, threats), physical (e.g., hitting, kicking), or social (e.g., exclusion) acts perpetrated by one or more students. It excludes mutual conflicts between peers of similar power or playful, consensual teasing [66,67].

### Data quality control

The Tigrigna language questionnaire was used to collect data. To ensure data quality, the original English questionnaire was translated into Tigrigna (the local language) through a rigorous process to ensure validity across cultures. First, two forward translations were combined into one draft. Then this draft was back-translated into English to ensure it matched the original concepts. An expert review panel comprising bilingual mental health professionals and a language expert assessed all versions for meaning, idioms, and ideas. Finally, the Tigrigna questionnaire was pretested two weeks before formal data collection among 43 students from non-participating schools, which confirmed that participants understood it well and that its internal reliability was acceptable (Cronbach’s α of 0.97).

Supervisors and data collectors completed a comprehensive two-day training program. During fieldwork, on-site supervision was implemented at all data collection points. Daily, the supervisors checked the completeness of the questionnaires.

### Data analysis

The data were checked, coded, and entered into Epi-Data software (Version 3.1), then exported to SPSS (Statistical Package for the Social Sciences, Version 25) for data analysis. Descriptive statistics (percentages, means, standard deviations) were used to characterize respondents’ socio-demographic attributes and other variables.

Binary logistic regression was used to determine the association between the independent variables and the outcome variable. Bivariate logistic regression was first performed to identify the associations between each independent variable and the outcome variable. Variables significant at p ≤ 0.25 in bivariate analyses were entered into multivariate logistic regression to adjust for confounding effects. Statistical significance was defined as p < 0.05, with adjusted odds ratios (AORs) and 95% confidence intervals (CIs) computed. The results are presented in tables, which display frequency distributions and summary statistics (e.g., means and percentages), as well as in supplementary figures.

We fitted a multivariable logistic regression model that included only significant variables from the bivariate analyses (p < 0.05) to evaluate the robustness of our results. The results are robust to changes in model specification because the direction and strength of the associations in this parsimonious model were consistent with those in the main model.

The model’s goodness of fit was assessed using the Hosmer-Lemeshow test, and the results indicated adequate fit (P = 0.273). The model showed significant explanatory power, with a Nagelkerke R² value of 0.578, and exceptional predictive accuracy, with an Area Under the Curve (AUC) of 0.905 (95% CI [0.88, 0.930], p < .001). Multicollinearity was also evaluated using variance inflation factors (VIFs), and all variables had VIFs below 5.

Interaction terms between sex and different trauma types were fitted in the model to examine effect modification. A statistically significant interaction of gender and contact sexual abuse was observed, which was further explored by stratified analyses. Two-tailed p < 0.05 was considered statistically significant for main effects, and the results were presented as Adjusted Odds Ratios (AORs) and 95% Confidence Intervals (CIs).

### Ethical considerations

Ethical clearance was obtained from Adigrat University, College of Medicine and Health Sciences, Health Research Ethics Review Committee, with the code number ADU.CMHS/HRERC/0009/2024. Additionally, a formal letter was received from the town Education Office, which was provided to the respective schools before the actual data collection.

Students were assured that their participation would not impact their academic performance, and confidentiality was strictly maintained. For students aged 18 and older, verbal informed consent was obtained. For students under 18, verbal informed consent was secured from their parents, and the students provided assent. Students were instructed to consult the trained and qualified facilitator for any study-related concerns. Privacy was maintained through spaced seating, preventing participants from viewing each other’s questionnaire responses.

## Results

### Socio-demographic characteristics of participants

All 608 students were assessed and found eligible based on the criteria; 599 completed the self-administered questionnaire, resulting in a response rate of 98.2%. Participants’ ages ranged from 15 to 24 years, with a mean age of 17.61 years (±1.63).

Out of the total respondents, 506 (84.5%) attended public schools. Most participants, 574 (95.8%), identified as members of the Tigrian ethnicity, and 545 (91.0%) followed the Orthodox faith. Approximately 385 participants (64.3%) lived with both parents. More than one-third of participants, 233 (38.9%), were in grade 9, while 179 (29.9%) reported having more than three close friends (Table 1).

**Table 1:** Distribution of socio-demographic characteristics of secondary school students in Adigrat Town, 2024, N = 599.

Variables	Category	Frequency	Percent
Gender	Male	255	42.6
	Female	344	57.4
Age	15-17 years	288	48.1
	18-24 Years	311	51.9
Family structure (living with)	Both parents	385	64.3
	Single parent	138	23.0
	Others*	76	12.7
Ethnicity	Tigrian	574	95.8
	Others**	25	4.2
Religion	Orthodox Christianity	545	91.0
	Catholic	43	7.2
	Others***	11	1.9
School type	Public	506	84.5
	Private	93	15.5
Grade	Nine	233	38.9
	Ten	126	21.0
	Eleven	117	19.5
	Twelve	123	20.5
Having close friends	None	125	20.9
	One	127	21.2
	Two	168	28.0
	Three or more	179	29.9
Residence	Urban	475	79.3
	Rural	124	20.7

**Note:** *Grandparents, Siblings, Uncles, **Amhara, Oromia, ***Muslim, Protestant.

### Behavioral characteristics of the participants

Substance use and physical activity levels were assessed among 599 secondary students. The findings revealed that 88.1% of participants reported ever using alcoholic beverages, while only 6.5% and 4.8% had ever used khat and tobacco, respectively. Current alcohol use was reported by 44.7% of students, and in terms of physical activity, 54.4% of students did not meet the recommended levels (Table 2).

**Table 2:** Distribution of behavioral characteristics among secondary school students in Adigrat Town, 2024, N = 599.

Variables	Categories	Frequency	Percent
Alcoholic beverages			
Ever use history	Yes	528	88.1
	No	71	11.9
Current use history	Yes	268	44.7
	No	331	55.3
Khat			
Ever use history	Yes	39	6.5
	No	560	93.5
Current use history	Yes	25	4.2
	No	574	95.8
Tobacco products			
Ever use history	Yes	29	4.8
	No	570	95.2
Current use history	Yes	18	3
	No	581	97
Hashish			
Ever use history	Yes	26	4.3
	No	573	95.7
Current use history	Yes	19	3.2
	No	580	96.8
Meeting recommended physical activity	Yes	273	45.6
	No	326	54.4

### Trauma exposure and psychosocial-related characteristics of the study participants

Among the 599 students surveyed, the most striking finding was the high prevalence of battlefield exposure: 84.1% reported combat experience or exposure to the battlefield. Physical violence was also widespread: 45.9% reported direct experiences, while 28.5% witnessed such events. Regarding sexual violence, 16.9% experienced non-contact sexual abuse, and 13.0% reported contact sexual abuse.

**Fig 1:**
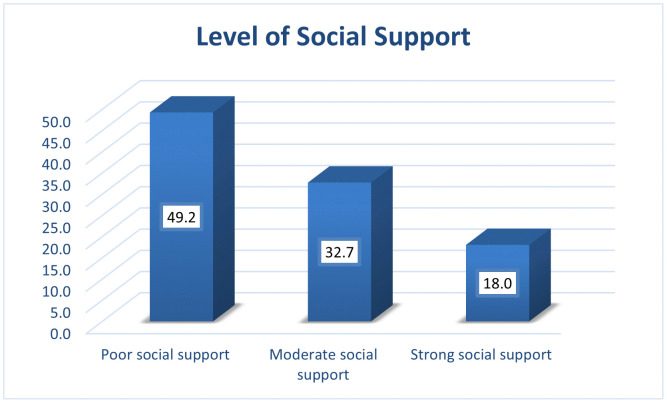
Distributions of perceived social support levels among secondary school students in Adigrat Town, Ethiopia, 2024, N = 599.

In our sample, 23.9% of participants said they had thought about suicide at some point in their lives. Additionally, 12.2% reported that they had attempted suicide at least once. In the preceding 12 months, 19.9% experienced suicidal ideation, and 9.7% had attempted suicide. Recent bullying (within the past 30 days) was reported by 20.0% of participants (Table 3). Additionally, 49.2% reported poor social support (Fig 1).

**Table 3:** Trauma exposure and psychosocial-related characteristics among secondary school students in Adigrat Town, 2024, N = 599.

Traumatic event variables	Category	Frequency	Percent
Experiencing the sudden death of a family member or someone close	Yes	139	23.2
	No	460	76.8
Non-contact sexual abuse	Yes	101	16.9
	No	498	83.1
Contact sexual abuse	Yes	78	13.0
	Yes	521	87.0
Experiencing physical violence	Yes	275	45.9
	No	324	54.1
Witnessing physical violence	Yes	171	28.5
	No	428	71.5
History of combat or exposure to a battlefield	Yes	504	84.1
	No	95	15.9
Lifetime suicidal ideation	Yes	143	23.9
	No	456	76.1
Suicidal ideation in the last 12 months	Yes	119	19.9
	No	480	80.1
A lifetime suicidal plan	Yes	73	12.2
	No	526	87.8
Suicidal plan in the last 12 months	Yes	54	9.0
Lifetime suicidal attempt	Yes	74	12.2
	No	526	87.8
Suicidal attempt in the last 12 months	Yes	47	7.8
	No	552	92.2
Bullied	Yes	120	20.0
	No	479	80.0
Depression	Yes	443	74.0
	No	156	26.0

### Prevalence of clinically significant anxiety symptoms

**Fig 2:**
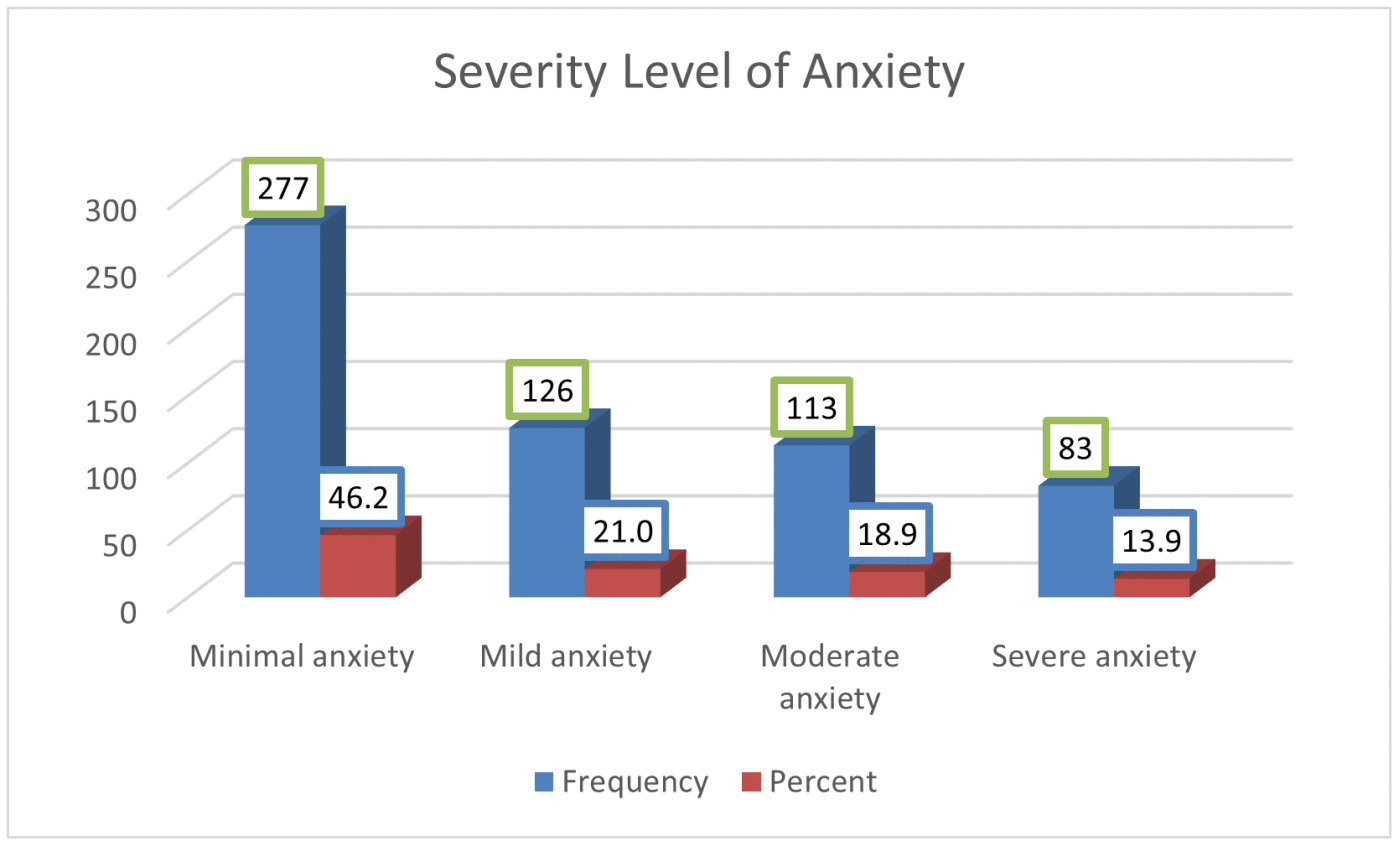
Distribution of anxiety severity level among secondary school students in Adigrat Town, Ethiopia, 2024, N = 599.

The overall magnitude of clinically significant anxiety symptoms among the study participants was 32.7%, with a 95% CI (28.9%, 36.5%). The sample included 344 females (57.4%) and 255 males (42.6%). Consistent with this distribution, the magnitude of anxiety was significantly higher among females (47.4%, 95% CI: 42.1%, 52.7%) compared to males (12.9%, 95% CI: 8.8%, 17.1%). Furthermore, almost one-seventh of the students were experiencing severe levels of anxiety symptoms, as shown in Fig 2.

### Factors associated with clinically significant anxiety symptoms

Multivariable logistic regression revealed that being female, being 18 or older, experiencing attacks, shootings, or stabbings, exposure to war, being bullied, having a history of contact sexual abuse, suicidal ideation, and depression were significantly associated with clinically significant anxiety symptoms (Table 4).

**Table 4:** Factors associated with clinically significant anxiety symptoms among secondary school students in Adigrat, 2024, N = 599.

Variables	Category	Anxiety		COR (95% CI)	AOR (95% CI)	P-value
		Yes	No			
Gender	Male	33	222	1	1	
	Female	163	181	6.06 (3.97, 9.24)	6.52 (3.70, 11.46)	0.000**
Age	15-17	59	229	1	1	
	18-24	137	174	3.06 (2.13, 4.40)	3.19 (1.60, 6.39)	0.001**
Family structure	With both parents	116	269	1	1	
	With a single parent	44	94	1.09 (0.71, 1.65)	0.82 (0.45, 1.48)	0.506
	Others	36	40	2.09 (1.27, 3.44)	0.99 (0.48, 2.03)	0.975
Grade	Nine	51	182	1	1	
	Ten	44	82	1.92 (1.19, 3.10)	1.21 (0.58, 2.50)	0.609
	Eleven	50	67	2.66 (1.65, 4.31)	0.90 (0.38, 2.12)	0.806
	Twelve	51	72	2.53 (1.57, 4.06)	0.71 (0.29, 1.70)	0.437
Social support	Strong	23	85	1	1	
	Moderate	61	135	1.67 (0.96, 2.90)	0.98 (0.46, 2.07)	0.955
	Poor	112	183	2.26 (1.35, 3.79)	1.19 (0.57, 2.45)	0.644
Current alcohol use	No	85	246	1	1	
	Yes	111	157	2.05 (1.45, 2.89)	0.99 (0.60, 1.62)	0.955
Sudden death of a family member or someone close	No	114	346	1	1	
	Yes	82	57	4.37 (2.93, 6.51)	1.12 (0.62, 2.05)	0.702
Experiencing physical violence	No	69	255	1	1	
	Yes	127	148	3.17 (2.22, 4.53)	2.25 (1.32, 3.82)	0.003*
Witnessing physical violence	No	106	322	1	1	
	Yes	90	81	3.38 (2.33, 4.90)	1.42 (0.81, 2.51)	0.224
History of combat or exposure to a battlefield	No	6	89	1	1	
	Yes	190	314	8.98 (3.85, 20.92)	5.05 (1.95, 13.12)	0.001**
Bullying experience	No	129	350	1	1	
	Yes	67	53	3.43 (2.27, 5.18)	2.26 (1.24, 4.11)	0.008*
Non-contact sexual abuse	No	142	356	1	1	
	Yes	54	47	2.88 (1.86, 4.46)	0.49 (0.21, 1.13)	0.094
Contact sexual abuse	No	143	378	1	1	
	Yes	53	25	5.60 (3.36, 9.36)	3.97 (1.58, 9.96)	0.003
History of suicide ideation	No	96	360	1	1	
	Yes	100	43	8.72 (5.72, 13.31)	4.01 (1.96, 8.24)	0.000**
History of plans for committing suicide	No	143	383	1	1	
	Yes	53	20	7.10 (4.10, 12.29)	1.16 (0.44, 3.04)	0.766
						
History of attempted suicide	No	142	383	1	1	
	Yes	54	20	7.28 (4.21, 12.60)	0.89 (0.33, 2.39)	0.823
Depression	No	111	45	1	1	
	Yes	85	358	10.39 (6.83, 15.80)	3.99 (2.32, 6.87)	0.000**

**Note:** 1.00 remained for the reference category, *significance level at p-value <0.05, ** significance level at p-value <=0.001.

**Abbreviations:** COR, Crude Odds Ratio; AOR, Adjusted Odds Ratio; CI, confidence interval.

Specifically, female students were 6.52 times more likely to develop anxiety compared to male students (AOR = 6.52, 95% CI: 3.70, 11.46). Students aged 18 years and older were 3.19 times more likely to experience anxiety compared to those younger than 18 years (AOR = 3.19, 95% CI: 1.60, 6.39). Students who had a history of combat or exposure to a battlefield were five times more likely to develop anxiety than those students without a history of combat or exposure to a battlefield (AOR = 5.05, 95% CI: 1.95, 13.12). Experiencing physical violence such as attacks, shootings, or stabbings increased the odds of anxiety by 2.25 times (AOR = 2.25, 95% CI: 1.32, 3.82). Students who had experienced bullying were 2.26 times more likely to have anxiety (AOR = 2.26, 95% CI: 1.24, 4.11). The history of contact sexual abuse elevated the likelihood of anxiety by a factor of 3.97 times (AOR = 3.97, 95% CI: 1.58, 9.96). Anxiety was 4.01 times more common in students with a history of suicide ideation (AOR = 4.01, 95% CI: 1.96, 8.24). The odds of developing anxiety were 3.99 times greater among students with depression than those without (AOR = 3.99, 95% CI: 2.32, 6.87).

Furthermore, Table 5 below presents the findings from testing for effect modification between gender and different types of traumas. Gender and contact sexual abuse were found to interact significantly (AOR = 2.79, 95% CI [1.85, 4.19], p < 0.001). Other interactions between trauma and gender did not show statistical significance. Stratified analyses were performed to interpret the significant interaction. The relationship between anxiety and contact sexual abuse was statistically significant for females (AOR = 4.11, 95% CI [1.31, 12.89], p = 0.015), but not for males (AOR = 2.20, 95% CI [0.38, 12.84], p = 0.381), suggesting a gender-specific effect in which females were more susceptible to anxiety after this particular trauma.

**Table 5:** Interaction effect between gender and trauma types on anxiety among secondary school students in Adigrat, 2024, N = 599.

Interaction term	COR (95% CI)	AOR (95% CI)	P-value
Gender*Contact sexual abuse	2.92 (2.13, 4.01)	2.79 (1.85, 4.19)	0.000
Gender*non-contact sexual abuse	1.92 (1.49, 2.47)	1.10 (0.78, 1.55)	0.604
Gender*Sudden death of a family member or friend	1.20 (1.02, 1.40)	1.16 (0.90, 1.50)	0.253
Gender*Experiencing physical violence	1.13 (0.96, 1.32)	0.94 (0.74, 1.18)	0.590
Gender*Witnessing physical violence	1.18 (1.01, 1.38)	1.05 (0.81, 1.36)	0.713
Gender*History of combat or exposure to a battlefield	0.293 (0.19, 0.44)	1.15 (0.86, 1.54)	0.355

**Note:** The model contained all the listed interaction terms and main effects. Male and female were coded as 0 and 1, respectively. A significant interaction suggests that the impact of the trauma on anxiety symptoms is different for men and women.

## Discussion

The high prevalence of clinically significant anxiety symptoms identified in this study indicates that nearly one in three (32.7%, 95% CI (28.9%, 36.5%) Tigrian secondary school students suffer from anxiety, highlighting the enduring mental health burden long after active hostilities have ended. When placed in context, this prevalence is significantly elevated. In non-conflict settings of Ethiopia, a systematic review among children and youth showed a prevalence of 0.5% to 23% [26], and a school-based study showed a prevalence of 25.05% [27]. Even within Tigray, the pre-war anxiety prevalence was 11.48% [28]. The current prevalence closely matches the 34.43% reported in a wartime survey of Tigrayan youth [28], despite differences in methods and timing. This underlines the strong and lasting psychological effects of the conflict.

Our finding aligns with the longitudinal research in Sierra Leone, which showed that exposure to war-related trauma strongly predicts ongoing mental health issues in young people even years after the conflict ended [68]. Furthermore, our result is consistent with the study conducted among conflict-affected Palestinian adolescents, which reported a prevalence of 30.9% [69]. All things considered, these findings indicate that Tigrian students continue to experience severe anxiety symptoms despite the official ceasefire agreement, which highlights the urgent need for focused, sustained mental health interventions in schools affected by war.

However, the prevalence in this study was slightly higher than that reported in Uganda (26.6%) [70], Afghanistan (23.9%) [22], and another study from Palestinian (23.7%) [23], and lower than estimates from Sudan (50.8%) [71], Woldia Town, Ethiopia (39.7%) [25], the Gaza Strip (38%) [20], and Syrian and Iraqi refugee youth (37%) [72]. These differences among countries affected by war are not surprising and should be interpreted with caution. Factors such as the tools used for assessment, the timing of evaluations relative to exposure to conflict, the nature and severity of the conflicts, and various sociocultural factors likely explain much of the observed variation.

Female students were 6.52 times more likely to develop anxiety than male students in this study. This result is consistent with other studies conducted in Palestine [22], Ethiopia [25], and India [36]. The higher vulnerability could be due to a variety of factors that are more common in females, including their vulnerability to gender-related trauma like rape and violence [73,74], as well as socio-economic factors like gender disparities in caregiving and economic conditions [75]. Other pressures associated with school performance, friendships, and sleep problems could also be contributing to higher levels of anxiety in girls [76]. In addition, the lack of mental health care and the stigma associated with mental illness could also be contributing to delays in seeking care [77].

Students aged 18 years and older were 3.19 times more likely to experience anxiety compared to those younger than 18 years. Other studies support this finding [22,39]. This could be better explained by the fact that the period of transition from adolescence to young adulthood is often associated with heightened anxiety levels as individuals navigate new challenges such as higher education, career choices, and financial independence [78,79].

Students who were exposed to traumatic events such as violent attacks or battlefields were much more likely to experience anxiety symptoms than those who were not exposed. This is consistent with the evidence showing that individuals who were exposed to violent conflicts experience greater levels of anxiety symptoms due to direct exposure to traumatic events, including the witnessing of violence or being forced to relocate [20,25,80]. Therefore, early identification and appropriate mental health care, as well as encouraging positive ways of coping, are especially important in a post-conflict environment [69,81].

Bullying victimization and mental health outcomes are significantly correlated, as evidenced by the finding that students who experienced bullying were 2.26 times more likely to have anxiety. This finding is consistent with previous research showing that exposure to bullying can have adverse psychological effects, such as an increase in anxiety symptoms [82,83]. Bullying-induced chronic stress may dysregulate the HPA axis, increasing susceptibility to anxiety disorders [84], while growing evidence also suggests that bullying could change brain structures involved in emotional regulation, such as the amygdala [85]. Moreover, victims of bullying often experience social withdrawal and negative self-perception, which further exacerbates anxiety symptoms [86]. Collectively, these mechanisms highlight how bullying impacts mental health through both physiological and psychological pathways.

Anxiety was about four times more common among students who had experienced contact sexual abuse compared to those who had not. This strong link supports earlier research indicating that sexual abuse is a major risk factor for anxiety disorders, including panic disorder, generalized anxiety disorder, and PTSD [87]. Furthermore, this finding aligns with evidence that women who experience childhood sexual abuse are more likely to develop internalizing disorders, like anxiety [88]. Neurobiological evidence suggests that trauma can disrupt the HPA axis, increasing stress reactivity and maintaining anxiety [18], along with social and cognitive factors like hypervigilance, stigma, and lack of support [89,90], might increase this vulnerability. These results highlight the need for gender-sensitive, trauma-informed treatments, such as cognitive-behavioral therapy (CBT) and eye movement desensitization and reprocessing (EMDR). These approaches have shown success in reducing anxiety for abuse survivors [91].

Students who have thought about suicide are 4.01 times more likely to experience anxiety. This aligns with studies that show a strong link between anxiety disorders and suicidal thoughts [92,93]. Anxiety symptoms, especially worry and trouble sleeping, can predict suicidal thoughts on their own, even after considering depression [92]. There is also evidence of a relationship between the severity of anxiety and suicidal behaviors [93]. These findings indicate that anxiety and suicidal thoughts may influence each other. Therefore, it is important to screen students who report suicidal thoughts for anxiety and vice versa, since dealing with both may require combined treatment.

Students with depression are almost four times more likely to develop anxiety symptoms compared to those without depression symptoms. This supports the established connection between anxiety and depression, as shown in the tripartite model, which points out shared negative feelings and unique traits of depression and anxiety [94]. Previous research shows significant overlap between the two conditions [94,95]. Since having both conditions often leads to worse treatment results and higher chances of relapse, it is crucial to identify overlapping symptoms early and focus on treatments that address common factors [93,94].

## Limitations and strengths of the study

The cross-sectional design of this study limits the ability to establish causal relationships between anxiety and its risk factors. However, this study provides significant preliminary insights for future longitudinal research.

The following aspects limit the generalizability of these findings. First, since the data were collected from secondary schools in Adigrat town, the results might not represent the whole Tigray region or other post-conflict areas. Second, the school-based sampling frame may limit the extent to which we can generalize our findings, as it leaves out-of-school youth who might be at greater risk for trauma exposure and anxiety. Third, the inclusion of students up to 24 years old may limit direct comparisons with studies done in typical high school populations. We should keep this potential selection bias in mind when interpreting the results.

Furthermore, self-reported data on trauma exposure (e.g., bullying, sexual abuse) may be influenced by recall or social desirability biases, leading to potential underreporting. To address this, in addition to using well-validated and standardized tools, participants’ anonymity was ensured, which strengthens the reliability of the responses.

## Conclusion

Nearly one-third of high school students were found to have clinically significant anxiety symptoms. This anxiety was significantly associated with being female, age 18 or older, direct exposure to battlefield or combat situations, physical violence, bullying, contact sexual abuse, and co-occurring suicidal ideation and depression.

These findings highlight the urgent need for targeted mental health programs in schools. Such initiatives should go beyond general support to include early screening for these specific risk factors and provide effective, trauma-informed treatments. For example, trauma-focused cognitive behavioral therapy can help address the unique psychological issues arising from war exposure. It’s also crucial to integrate effective suicide risk assessments and depression management. This approach is necessary to reduce the long-term psychological impact on this vulnerable generation.

## Supporting information

S1 Table Completed STROBE checklist for cross-sectional studies. (DOC)

S1 Data Data sets for anxiety among secondary school students in the war-torn Tigray (n = 599). (SAV)

## References

[1] El-Khodary B, Samara M. Effectiveness of a School-Based Intervention on the Students’ Mental Health After Exposure to War-Related Trauma. Front Psychiatry. 2020;10:1031. doi: 10.3389/fpsyt.2019.01031 32273852 PMC7113368

[2] Chrisman AK, Dougherty JG. Mass trauma: disasters, terrorism, and war. Child Adolesc Psychiatr Clin N Am. 2014;23(2):257–79, viii. doi: 10.1016/j.chc.2013.12.004 24656579

[3] Betancourt TS, Keegan K, Farrar J, Brennan RT. The intergenerational impact of war on mental health and psychosocial wellbeing: lessons from the longitudinal study of war-affected youth in Sierra Leone. Confl Health. 2020;14:62. doi: 10.1186/s13031-020-00308-7 32884581 PMC7461150

[4] Charlson F, van Ommeren M, Flaxman A, Cornett J, Whiteford H, Saxena S. New WHO prevalence estimates of mental disorders in conflict settings: a systematic review and meta-analysis. Lancet. 2019;394(10194):240–8. doi: 10.1016/S0140-6736(19)30934-1 31200992 PMC6657025

[5] Carpiniello B. The Mental Health Costs of Armed Conflicts-A Review of Systematic Reviews Conducted on Refugees, Asylum-Seekers and People Living in War Zones. Int J Environ Res Public Health. 2023;20(4):2840. doi: 10.3390/ijerph20042840 36833537 PMC9957523

[6] Barlow DH. Anxiety and its disorders: The nature and treatment of anxiety and panic. Guilford press. 2004.

[7] John-Henderson NA, Williams SE, Brindle RC, Ginty AT. Changes in sleep quality and levels of psychological distress during the adaptation to university: The role of childhood adversity. Br J Psychol. 2018;109(4):694–707. doi: 10.1111/bjop.12314 29799113

[8] Kessler RC, Berglund P, Demler O, Jin R, Merikangas KR, Walters EE. Lifetime prevalence and age-of-onset distributions of DSM-IV disorders in the National Comorbidity Survey Replication. Arch Gen Psychiatry. 2005;62(6):593–602. doi: 10.1001/archpsyc.62.6.593 15939837

[9] Kessler RC, Petukhova M, Sampson NA, Zaslavsky AM, Wittchen H-U. Twelve-month and lifetime prevalence and lifetime morbid risk of anxiety and mood disorders in the United States. Int J Methods Psychiatr Res. 2012;21(3):169–84. doi: 10.1002/mpr.1359 22865617 PMC4005415

[10] Banyard VL, Cantor EN. Adjustment to College Among Trauma Survivors: An Exploratory Study of Resilience. csd. 2004;45(2):207–21. doi: 10.1353/csd.2004.0017

[11] McLaughlin KA, Conron KJ, Koenen KC, Gilman SE. Childhood adversity, adult stressful life events, and risk of past-year psychiatric disorder: a test of the stress sensitization hypothesis in a population-based sample of adults. Psychol Med. 2010;40(10):1647–58. doi: 10.1017/S0033291709992121 20018126 PMC2891275

[12] Murphy F, Nasa A, Cullinane D, Raajakesary K, Gazzaz A, Sooknarine V, et al. Childhood Trauma, the HPA Axis and Psychiatric Illnesses: A Targeted Literature Synthesis. Front Psychiatry. 2022;13:748372. doi: 10.3389/fpsyt.2022.748372 35599780 PMC9120425

[13] Miller KE, Rasmussen A. War exposure, daily stressors, and mental health in conflict and post-conflict settings: bridging the divide between trauma-focused and psychosocial frameworks. Soc Sci Med. 2010;70(1):7–16. doi: 10.1016/j.socscimed.2009.09.029 19854552

[14] Cross D, Fani N, Powers A, Bradley B. Neurobiological Development in the Context of Childhood Trauma. Clin Psychol (New York). 2017;24(2):111–24. doi: 10.1111/cpsp.12198 30906116 PMC6428430

[15] De Bellis MD, AZ AB. The biological effects of childhood trauma. Child and Adolescent Psychiatric Clinics of North America. 2014;23(2):185. 24656576 10.1016/j.chc.2014.01.002 PMC3968319

[16] Juruena MF, Eror F, Cleare AJ, Young AH. The role of early life stress in HPA axis and anxiety. Anxiety disorders: Rethinking and understanding recent discoveries. 2020. 10.1007/978-981-32-9705-0_9 32002927

[17] Pervanidou P, Chrousos GP. Posttraumatic stress disorder in children and adolescents: neuroendocrine perspectives. Sci Signal. 2012;5(245):pt6. doi: 10.1126/scisignal.2003327 23047921

[18] Heim C, Nemeroff CB. The role of childhood trauma in the neurobiology of mood and anxiety disorders: preclinical and clinical studies. Biol Psychiatry. 2001;49(12):1023–39. doi: 10.1016/s0006-3223(01)01157-x 11430844

[19] Karam EG, Fayyad J, Karam AN, Melhem N, Mneimneh Z, Dimassi H, et al. Outcome of depression and anxiety after war: a prospective epidemiologic study of children and adolescents. J Trauma Stress. 2014;27(2):192–9. doi: 10.1002/jts.21895 24740870

[20] Hinchey LM-E, Nashef R, Bazzi C, Gorski K, Javanbakht A. The longitudinal impact of war exposure on psychopathology in Syrian and Iraqi refugee youth. Int J Soc Psychiatry. 2023;69(7):1833–6. doi: 10.1177/00207640231177829 37278010

[21] Elbedour S, Onwuegbuzie AJ, Ghannam J, Whitcome JA, Abu Hein F. Post-traumatic stress disorder, depression, and anxiety among Gaza Strip adolescents in the wake of the second Uprising (Intifada). Child Abuse Negl. 2007;31(7):719–29. doi: 10.1016/j.chiabu.2005.09.006 17631959

[22] Kolltveit S, Lange-Nielsen II, Thabet AAM, Dyregrov A, Pallesen S, Johnsen TB, et al. Risk factors for PTSD, anxiety, and depression among adolescents in Gaza. J Trauma Stress. 2012;25(2):164–70. doi: 10.1002/jts.21680 22522730

[23] Panter-Brick C, Eggerman M, Gonzalez V, Safdar S. Violence, suffering, and mental health in Afghanistan: a school-based survey. The Lancet. 2009;374(9692):807–16. 10.1016/S0140-6736(09)61080-1 PMC2748901 19699514

[24] Pat-Horenczyk R, Abramovitz R, Peled O, Brom D, Daie A, Chemtob CM. Adolescent exposure to recurrent terrorism in Israel: posttraumatic distress and functional impairment. Am J Orthopsychiatry. 2007;77(1):76–85. doi: 10.1037/0002-9432.77.1.76 17352588

[25] Kassa MA, Anbesaw T, Nakie G, Melkam M, Azmeraw M, Semagn EG, et al. Investigating war trauma, its effects, and associated risk factors on anxiety among high school students in Woldia town, northeast Ethiopia, 2022. Front Psychiatry. 2024;15:1368285. doi: 10.3389/fpsyt.2024.1368285 39056017 PMC11270624

[26] Berhanu Boru B, Yonas Deressa G. A Systematic Review and Meta-Analysis of Anxiety among Children and Youth in Ethiopia. J Depress Anxiety Disord. 2021;3(2). doi: 10.36959/362/479

[27] Gebreegziabher ZA, Eristu R, Molla A. Determinants of adolescents’ depression, anxiety, and somatic symptoms in Northwest Ethiopia: A non-recursive structural equation modeling. PLoS One. 2024;19(4):e0281571. doi: 10.1371/journal.pone.0281571 38598540 PMC11006201

[28] Favara M, Hittmeyer A, Porter C, Singhal S, Woldehanna T. Young people, mental health, and civil conflict: Preliminary findings from Ethiopia’s Tigray region. Psychiatry Research Communications. 2022;2(1):100025. doi: 10.1016/j.psycom.2022.100025

[29] Gesesew H, Kebede H, Berhe K, Fauk N, Ward P. Perilous medicine in Tigray: a systematic review. Confl Health. 2023;17(1):26. doi: 10.1186/s13031-023-00524-x 37254199 PMC10228460

[30] Yemane A, Teka H, Tesfay F, Gidey H, Tekle A, Tadesse Y, et al. Obstetrics and gynaecology in an Ethiopian war zone. BJOG. 2022;129(12):1953–6. doi: 10.1111/1471-0528.17238 35611573

[31] Devi S. Tigray atrocities compounded by lack of health care. Lancet. 2021;397(10282):1336. doi: 10.1016/S0140-6736(21)00825-4 33838749

[32] Gesesew H, Berhane K, Siraj ES, Siraj D, Gebregziabher M, Gebre YG, et al. The impact of war on the health system of the Tigray region in Ethiopia: an assessment. BMJ Glob Health. 2021;6(11):e007328. doi: 10.1136/bmjgh-2021-007328 34815244 PMC8611430

[33] Fisseha G, Gebrehiwot TG, Gebremichael MW, Wahdey S, Meles GG, Gezae KE, et al. War-related sexual and gender-based violence in Tigray, Northern Ethiopia: a community-based study. BMJ Glob Health. 2023;8(7):e010270. doi: 10.1136/bmjgh-2022-010270 37479499 PMC10364179

[34] Amendola S, von Wyl A, Volken T, Zysset A, Huber M, Dratva J. A Longitudinal Study on Generalized Anxiety Among University Students During the First Wave of the COVID-19 Pandemic in Switzerland. Front Psychol. 2021;12:643171. doi: 10.3389/fpsyg.2021.643171 33776867 PMC7990874

[35] Conley CS, Shapiro JB, Huguenel BM, Kirsch AC. Navigating the College Years: Developmental Trajectories and Gender Differences in Psychological Functioning, Cognitive-Affective Strategies, and Social Well-Being. Emerging Adulthood. 2018;8(2):103–17. doi: 10.1177/2167696818791603

[36] Sandal RK, Goel NK, Sharma MK, Bakshi RK, Singh N, Kumar D. Prevalence of Depression, Anxiety and Stress among school going adolescent in Chandigarh. J Family Med Prim Care. 2017;6(2):405–10. doi: 10.4103/2249-4863.219988 29302555 PMC5749094

[37] Tramonte L, Willms D. The prevalence of anxiety among middle and secondary school students in Canada. Can J Public Health. 2010;101 Suppl 3(Suppl 3):S19-22. doi: 10.1007/BF03403977 21416799 PMC6974041

[38] Malak MZ, Khalifeh AH. Anxiety and depression among school students in Jordan: Prevalence, risk factors, and predictors. Perspect Psychiatr Care. 2018;54(2):242–50. doi: 10.1111/ppc.12229 28617949

[39] Osborn TL, Venturo-Conerly KE, Wasil AR, Schleider JL, Weisz JR. Depression and Anxiety Symptoms, Social Support, and Demographic Factors Among Kenyan High School Students. J Child Fam Stud. 2019;29(5):1432–43. doi: 10.1007/s10826-019-01646-8

[40] Mkhize M, van der Westhuizen C, Sorsdahl K. Prevalence and factors associated with depression and anxiety among young school-going adolescents in the Western Cape Province of South Africa. Compr Psychiatry. 2024;131:152469. doi: 10.1016/j.comppsych.2024.152469 38461564

[41] Gülsen A, Uygur B. Psychological Features of Smokers. Respir Care. 2018;63(12):1492–7. doi: 10.4187/respcare.06287 30065079

[42] Arnett JJ. Emerging adulthood. A theory of development from the late teens through the twenties. Am Psychol. 2000;55(5):469–80. doi: 10.1037/0003-066x.55.5.469 10842426

[43] Rice F, van den Bree MBM, Thapar A. A population-based study of anxiety as a precursor for depression in childhood and adolescence. BMC Psychiatry. 2004;4:43. doi: 10.1186/1471-244X-4-43 15596007 PMC545489

[44] Cifrodelli M, Rogante E, Moschillo A, Longhini L, Trocchia MA, Erbuto D, et al. The role of suicide severity in the association between anxiety symptoms and suicidal ideation: a mediation analysis. Eur Psychiatr. 2024;67(S1):S781–S781. doi: 10.1192/j.eurpsy.2024.1627 39120957

[45] Bronfenbrenner U. The ecology of human development: experiments by nature and design. Harvard University Press. 1979.

[46] Lazarus R, Folkman S. Stress, appraisal, and coping. Springer Publishing Company, Inc. 1984.

[47] Ganzel BL, Morris PA, Wethington E. Allostasis and the human brain: Integrating models of stress from the social and life sciences. Psychol Rev. 2010;117(1):134–74. doi: 10.1037/a0017773 20063966 PMC2808193

[48] Betancourt TS, Khan KT. The mental health of children affected by armed conflict: protective processes and pathways to resilience. Int Rev Psychiatry. 2008;20(3):317–28. doi: 10.1080/09540260802090363 18569183 PMC2613765

[49] Olson JA, Janoff-Bulman R. Shattered Assumptions: Towards a New Psychology of Trauma. New York: Free Press. 1992.

[50] Tol WA, Song S, Jordans MJD. Annual Research Review: Resilience and mental health in children and adolescents living in areas of armed conflict--a systematic review of findings in low- and middle-income countries. J Child Psychol Psychiatry. 2013;54(4):445–60. doi: 10.1111/jcpp.12053 23414226

[51] Manzar MD, Alghadir AH, Anwer S, Alqahtani M, Salahuddin M, Addo HA, et al. Psychometric Properties of the General Anxiety Disorders-7 Scale Using Categorical Data Methods: A Study in a Sample of University Attending Ethiopian Young Adults. Neuropsychiatr Dis Treat [Internet]. 2021 2021; 17:[893-903 pp.]. Available from: http://europepmc.org/abstract/MED/33790558 10.2147/NDT.S295912 PMC7997591 33790558

[52] Spitzer RL, Kroenke K, Williams JBW, Löwe B. A brief measure for assessing generalized anxiety disorder: the GAD-7. Arch Intern Med. 2006;166(10):1092–7. doi: 10.1001/archinte.166.10.1092 16717171

[53] Plummer F, Manea L, Trepel D, McMillan D. Screening for anxiety disorders with the GAD-7 and GAD-2: a systematic review and diagnostic metaanalysis. Gen Hosp Psychiatry. 2016;39:24–31. doi: 10.1016/j.genhosppsych.2015.11.005 26719105

[54] Gryczynski J, Kelly SM, Mitchell SG, Kirk A, O’Grady KE, Schwartz RP. Validation and performance of the Alcohol, Smoking and Substance Involvement Screening Test (ASSIST) among adolescent primary care patients. Addiction. 2015;110(2):240–7. 25311148 10.1111/add.12767 PMC4301997

[55] Humeniuk R, Henry-Edwards S, Ali R, Poznyak V, Monteiro MG. The Alcohol, Smoking and Substance Involvement Screening Test (ASSIST): Manual for Use in Primary Care. 2010.

[56] Humeniuk R, Ali R, Babor TF, Farrell M, Formigoni ML, Jittiwutikarn J, et al. Validation of the Alcohol, Smoking And Substance Involvement Screening Test (ASSIST). Addiction. 2008;103(6):1039–47. doi: 10.1111/j.1360-0443.2007.02114.x 18373724

[57] Bull FC, Al-Ansari SS, Biddle S, Borodulin K, Buman MP, Cardon G, et al. World Health Organization 2020 guidelines on physical activity and sedentary behaviour. Br J Sports Med. 2020;54(24):1451–62. doi: 10.1136/bjsports-2020-102955 33239350 PMC7719906

[58] Zolotor AJ, Runyan DK, Dunne MP, Jain D, Péturs HR, Ramirez C, et al. ISPCAN Child Abuse Screening Tool Children’s Version (ICAST-C): Instrument development and multi-national pilot testing. Child Abuse Negl. 2009;33(11):833–41. doi: 10.1016/j.chiabu.2009.09.004 19857897

[59] Kessler RC, Ustün TB. The World Mental Health (WMH) Survey Initiative Version of the World Health Organization (WHO) Composite International Diagnostic Interview (CIDI). Int J Methods Psychiatr Res. 2004;13(2):93–121. doi: 10.1002/mpr.168 15297906 PMC6878592

[60] Organization WH, Control CfD, Prevention. Global school-based student health survey (GSHS). 2013.

[61] Weathers F, Litz B, Keane T, Palmieri P, Marx B, Schnurr P. The PTSD Checklist for DSM-5 with Life Events Checklist for DSM-5 and Criterion A. A Measurement Instrument; National Center for PTSD: Baltimore, MD, USA. 2013.

[62] Kroenke K, Spitzer RL, Williams JB. The PHQ-9: validity of a brief depression severity measure. J Gen Intern Med. 2001;16(9):606–13. doi: 10.1046/j.1525-1497.2001.016009606.x 11556941 PMC1495268

[63] Richardson LP, McCauley E, Grossman DC, McCarty CA, Richards J, Russo JE, et al. Evaluation of the Patient Health Questionnaire-9 Item for detecting major depression among adolescents. Pediatrics. 2010;126(6):1117–23. doi: 10.1542/peds.2010-0852 21041282 PMC3217785

[64] Abiola T, Udofia O, Zakari M. Psychometric properties of the 3-item oslo social support scale among clinical students of Bayero University Kano, Nigeria. Malaysian Journal of Psychiatry. 2013;22(2):32–41.

[65] Kocalevent R-D, Berg L, Beutel ME, Hinz A, Zenger M, Härter M, et al. Social support in the general population: standardization of the Oslo social support scale (OSSS-3). BMC Psychol. 2018;6(1):31. doi: 10.1186/s40359-018-0249-9 30016997 PMC6050647

[66] Schneider SK, O’Donnell L, Stueve A, Coulter RWS. Cyberbullying, school bullying, and psychological distress: a regional census of high school students. Am J Public Health. 2012;102(1):171–7. doi: 10.2105/AJPH.2011.300308 22095343 PMC3490574

[67] Gebremedhin HT, Bifftu BB, Lebessa MT, Weldeyes AZ, Gebru TT, Petrucka P. Prevalence and Associated Factors of Psychological Distress Among Secondary School Students in Mekelle City, Tigray Region, Ethiopia: Cross-Sectional Study. Psychol Res Behav Manag. 2020;13:473–80. doi: 10.2147/PRBM.S252779 32547269 PMC7250291

[68] Betancourt TS, McBain R, Newnham EA, Brennan RT. Trajectories of internalizing problems in war-affected Sierra Leonean youth: examining conflict and postconflict factors. Child Dev. 2013;84(2):455–70. doi: 10.1111/j.1467-8624.2012.01861.x 23002719 PMC3656826

[69] Thabet A, Elhelou M, Vostanis P. Prevalence of PTSD, depression, and anxiety among orphaned children in the Gaza Strip. EC Paediatr. 2017;5(6):159–69.

[70] Abbo C, Kinyanda E, Kizza RB, Levin J, Ndyanabangi S, Stein DJ. Prevalence, comorbidity and predictors of anxiety disorders in children and adolescents in rural north-eastern Uganda. Child and adolescent psychiatry and mental health. 2013;7:1–11. 23841918 10.1186/1753-2000-7-21 PMC3710504

[71] Awad MH, Elmutasim M, Mohamed MM, Hemmeda L. Generalized anxiety disorder and associated factors among Sudanese adolescents during the Sudan Army conflict: A cross sectional study. Glob Epidemiol. 2025;9:100190. doi: 10.1016/j.gloepi.2025.100190 40061908 PMC11889574

[72] Wagner G, Glick P, Khammash U, Shaheen M, Brown R, Goutam P. Exposure to violence and its relationship to mental health among young people in Palestine. Eastern Mediterranean Health Journal. 2020;26(2):189–97. 32141597 10.26719/2020.26.2.189

[73] Donner NC, Lowry CA. Sex differences in anxiety and emotional behavior. Pflugers Arch. 2013;465(5):601–26. doi: 10.1007/s00424-013-1271-7 23588380 PMC3805826

[74] Maeng LY, Milad MR. Sex Differences in Anxiety Disorders: Gonadal Hormone Interactions with Pathophysiology, Neurobiology, and Treatment. Sex Differences in the Central Nervous System. Elsevier. 2016. p. 53–75. doi: 10.1016/b978-0-12-802114-9.00003-2

[75] Ward L. Worry-YING and Worry-YANG: A Critical Feminist Study with Women Who Worry. Issues Ment Health Nurs. 2024;45(7):715–23. doi: 10.1080/01612840.2024.2346591 38901022

[76] Bao C, Han L. Gender difference in anxiety and related factors among adolescents. Front Public Health. 2025;12:1410086. doi: 10.3389/fpubh.2024.1410086 39830180 PMC11738925

[77] Lim AG, Stock L, Shwe Oo EK, Jutte DP. Trauma and mental health of medics in eastern Myanmar’s conflict zones: a cross-sectional and mixed methods investigation. Confl Health. 2013;7(1):15. doi: 10.1186/1752-1505-7-15 23899166 PMC3750555

[78] Ichsan NANIMI, Ni’matuzahroh N. What causes anxiety in emerging adulthood?: A systematic review. International Journal of Scientific Research and Management. 2024;12(07):48–55.

[79] Wang S, Chong ZY, Zhang C, Xu W. Longitudinal Associations Between Anxiety and Depressive Symptoms in Adolescence, Early Adulthood, and Old Age: Cross-Lagged Panel Network Analyses. Depress Anxiety. 2024;2024:6205475. doi: 10.1155/da/6205475 40226714 PMC11919059

[80] Aldabbour B, Abuabada A, Lahlouh A, Halimy M, Elamassie S, Sammour AA-K, et al. Psychological impacts of the Gaza war on Palestinian young adults: a cross-sectional study of depression, anxiety, stress, and PTSD symptoms. BMC Psychol. 2024;12(1):696. doi: 10.1186/s40359-024-02188-5 39593100 PMC11600870

[81] Urbański PK, Schroeder K, Nadolska A, Wilski M. Symptoms of depression and anxiety among Ukrainian children displaced to Poland following the outbreak of the Russo-Ukrainian war: Associations with coping strategies and resilience. Appl Psychol Health Well Being. 2024;16(3):851–67. doi: 10.1111/aphw.12510 37974535

[82] Modecki KL, Minchin J, Harbaugh AG, Guerra NG, Runions KC. Bullying prevalence across contexts: a meta-analysis measuring cyber and traditional bullying. J Adolesc Health. 2014;55(5):602–11. doi: 10.1016/j.jadohealth.2014.06.007 25168105

[83] Gong Z, Reinhardt JD, Han Z, Ba Z, Lei S. Associations between school bullying and anxiety in children and adolescents from an ethnic autonomous county in China. Psychiatry Res. 2022;314:114649. doi: 10.1016/j.psychres.2022.114649 35643051

[84] Vaillancourt T, Hymel S, McDougall P. The Biological Underpinnings of Peer Victimization: Understanding Why and How the Effects of Bullying Can Last a Lifetime. Theory Into Practice. 2013;52(4):241–8. doi: 10.1080/00405841.2013.829726

[85] Teicher MH, Samson JA, Anderson CM, Ohashi K. The effects of childhood maltreatment on brain structure, function and connectivity. Nat Rev Neurosci. 2016;17(10):652–66. doi: 10.1038/nrn.2016.111 27640984

[86] Hawker DS, Boulton MJ. Twenty years’ research on peer victimization and psychosocial maladjustment: a meta-analytic review of cross-sectional studies. J Child Psychol Psychiatry. 2000;41(4):441–55. doi: 10.1111/1469-7610.00629 10836674

[87] Kessler RC, Aguilar-Gaxiola S, Alonso J, Benjet C, Bromet EJ, Cardoso G, et al. Trauma and PTSD in the WHO World Mental Health Surveys. Eur J Psychotraumatol. 2017;8(sup5):1353383. doi: 10.1080/20008198.2017.1353383 29075426 PMC5632781

[88] Amado BG, Arce R, Herraiz A. Psychological injury in victims of child sexual abuse: A meta-analytic review. Psychosocial Intervention. 2015;24(1):49–62. doi: 10.1016/j.psi.2015.03.002

[89] Ehlers A, Clark DM. A cognitive model of posttraumatic stress disorder. Behav Res Ther. 2000;38(4):319–45. doi: 10.1016/s0005-7967(99)00123-0 10761279

[90] Ullman SE, Brecklin LR. Sexual assault history and suicidal behavior in a national sample of women. Suicide Life Threat Behav. 2002;32(2):117–30. doi: 10.1521/suli.32.2.117.24398 12079028

[91] Bisson J. Psychological therapies for chronic PTSD in adults (review). Cochrane Review. 2013. 10.1002/14651858.CD003388.pub4 PMC6991463 24338345

[92] Busby Grant J, Batterham PJ, McCallum SM, Werner-Seidler A, Calear AL. Specific anxiety and depression symptoms are risk factors for the onset of suicidal ideation and suicide attempts in youth. J Affect Disord. 2023;327:299–305. doi: 10.1016/j.jad.2023.02.024 36764362

[93] Li J, Zhang Y, Siu Man Chan B, Tan SN, Lu J, Luo X, et al. Associations between anxiety, depression, and risk of suicidal behaviors in Chinese medical college students. Front Psychiatry. 2022;13:1012298. doi: 10.3389/fpsyt.2022.1012298 36532186 PMC9757065

[94] Cassady JC, Pierson EE, Starling JM. Predicting student depression with measures of general and academic anxieties. Frontiers in Education. Frontiers Media SA. 2019.

[95] Li W, Zhao Z, Chen D, Peng Y, Lu Z. Prevalence and associated factors of depression and anxiety symptoms among college students: a systematic review and meta-analysis. J Child Psychol Psychiatry. 2022;63(11):1222–30. doi: 10.1111/jcpp.13606 35297041

