# Supplementary material for: Anxiety among secondary school students in the war-torn Tigray, Ethiopia, 2024: A call for action
Source: PLOS Ment Health. 2026 Mar 18;3(3):e0000526. doi: 10.1371/journal.pmen.0000526 (PMC12998859; doi:10.1371/journal.pmen.0000526)
Supplement: S1 Table — (DOC) [file pmen.0000526.s001.doc]

S1 Table: Completed STROBE checklist for cross-sectional studies

This checklist was elaborated using formal items recommended for cross-sectional studies from the STROBE statement (https://www.strobe-statement.org).

|  | Item No | Recommendation | Respected? | Comments and quotes | |
| --- | --- | --- | --- | --- | --- |
| **Title and abstract** | 1 | (*a*) Indicate the study’s design with a commonly used term in the title or the abstract | Yes | | The study design is described in the Methods section of the abstract.  “A school-based cross-sectional study design was employed.” Page 3, Line 43 |
|  |  | (*b*) Provide in the abstract an informative and balanced summary of what was done and what was found | Yes | | This information is provided in the study abstract, including the study's objective, methods, results, and conclusions. |
| Introduction | | |  | |  |
| Background/rationale | 2 | Explain the scientific background and rationale for the investigation being reported. | Yes | | The rationale and existing literature are presented in the introduction section. |
| Objectives | 3 | State-specific objectives, including any prespecified hypotheses | Yes | | The specific goals and objectives are outlined in a statement at the end of the introduction.  “This pre-registered cross-sectional study, conducted in 2024, aimed to fill this gap by assessing the prevalence of anxiety and its associated factors among secondary school students in the Tigray Region.” Page 6, Lines 139-141 |
| Methods | | |  | |  |
| Study design | 4 | Present key elements of study design early in the paper | Yes | | Study design is outlined in the first subsection of Methods. All key elements are described in the methods.  “A school-based cross-sectional study was conducted from November 1 to 30, 2024, in Adigrat Town, one of the zonal capitals of the Tigray Region in northern Ethiopia.” Page 9, Lines 158-159 |
| Setting | 5 | Describe the setting, locations, and relevant dates, including periods of recruitment, exposure, follow-up, and data collection | Yes | | Settings, locations, and recruitment dates are thoroughly described in the method section under the "Study design, setting, and period” and “Study participants” headings on page 9. |
| Participants | 6 | (*a*) Give the eligibility criteria, and the sources and methods of selection of participants | Yes | | The source population, eligibility criteria, and selection process were described in the method subsections of the “Study participants” and “Sample size and sampling techniques” sections. Pages 9 and 10 |
| Variables | 7 | Clearly define all outcomes, exposures, predictors, potential confounders, and effect modifiers. Give diagnostic criteria, if applicable | Yes | | All variables are defined in the "Data collection and instruments" and "Operational definition" sections (pages 10-12, lines 190-241).  The diagnostic criteria for anxiety and depression are clearly outlined in the "Operational definition" section (page 12, lines 233-236).  Potential confounders and the method for controlling them, multivariate adjustment, are described in the "Data analysis" section (page 13, lines 262-264).  The assessment of effect modification by sex is detailed in the "Data analysis" section (page 14, lines 277-281). |
| Data sources/ measurement | 8* | For each variable of interest, give sources of data and details of methods of assessment (measurement). Describe comparability of assessment methods if there is more than one group | Yes | | All data were primary and collected using a structured, self-administered questionnaire. The source and assessment method for each variable are described in the “Data collection and instruments” subsection of the Methods section, spanning pages 10 to 12, lines 190 to 231.  Since this is a single population study, all participants received the same assessment methods. This guarantees that the comparisons are consistent. |
| Bias | 9 | Describe any efforts to address potential sources of bias | Yes | | To address potential sources of bias, various strategies were used. A stratified simple random sampling design improved representativeness and reduced selection bias (page 10, line 177). Validated tools and standard data-collection methods reduced measurement bias (pages 10-12, lines 190-231). Participant anonymity was stressed to limit social desirability bias (page 15, lines 291-292). Potential confounders were managed using multivariable logistic regression (page 13, lines 262-264). |
| Study size | 10 | Explain how the study size was arrived at | Yes | | The sample size was calculated using the single population proportion formula. It assumed an anxiety prevalence (p) of 39.7% from a prior study on adolescents affected by conflict, a 95% confidence level (Z=1.96), and a 5% margin of error (d). The initial estimate of 369 was adjusted for a design effect of 1.5 and a 10% non-response rate, resulting in a final required sample of 608 participants (page 9, lines 171-176). |
| Quantitative variables | 11 | Explain how quantitative variables were handled in the analyses. If applicable, describe which groupings were chosen and why | Yes | | In the analyses, researchers handled quantitative variables as follows: continuous scale scores, such as GAD-7, PHQ-9, and OSS-3, were analysed as categorical variables using standard clinical or validation-based cut-off points for binary logistic regression; for example, GAD-7 scores of 10 or higher indicated anxiety. Sociodemographic variables, such as age, were divided into meaningful groups, such as 15 to 17 years and 18 to 19 years, based on developmental stages and for clinical understanding. The main groupings and cut-offs were selected in advance, based on validated international and Ethiopian standards, to ensure clinical relevance and consistency with earlier studies. More details can be found in the “Data collection and instruments” and "Data analysis" sections on pages 10-12. |
| Statistical methods | 12 | (*a*) Describe all statistical methods, including those used to control for confounding | Yes | | The statistical methods are explained in detail in the "Data analysis" subsection. In summary, we conducted analyses using SPSS version 25. Descriptive statistics were calculated for all variables. We evaluated the relationship between the independent variables and the binary outcome (anxiety: GAD-7 ≥ 10) using binary logistic regression. To account for confounding, we followed a two-step analytical method. First, bivariate logistic regression identified candidate variables (p ≤ 0.25). Then, we included those in a multivariable logistic regression model to generate adjusted odds ratios (AORs) with 95% confidence intervals. We checked model fit using the Hosmer-Lemeshow test and Nagelkerke R². We also assessed predictive accuracy using AUC and multicollinearity using VIFs. To test for effect modification, we examined interaction terms (e.g., sex*trauma) and performed stratified analyses when interactions were significant (pages 13-14). |
|  |  | (*b*) Describe any methods used to examine subgroups and interactions | Yes | | Effect modification was assessed by including interaction terms, such as sex and trauma exposure, in regression models. Significant interactions led to stratified analyses that reported subgroup-specific associations (page 14, lines 277-281). |
|  |  | (*c*) Explain how missing data were addressed | Yes | | The electronic data entry system (EpiData) used built-in validation and required-field checks to prevent incomplete responses. As a result, the final dataset had no missing values, and data imputation was not needed (page 13, line 256) |
|  |  | (*d*) If applicable, describe analytical methods taking account of sampling strategy | N/A | | Non applicable |
|  |  | (*e*) Describe any sensitivity analyses | Yes | | Sensitivity analysis was conducted to assess the robustness of the findings and is described in the “Data analysis” subsection of the methods (page 14, lines 268-271). |
| Results | | |  | |  |
| Participants | 13* | (a) Report numbers of individuals at each stage of study—eg numbers potentially eligible, examined for eligibility, confirmed eligible, included in the study, completing follow-up, and analysed | Yes | | This is explained at the start of the results section.  “All 608 students were assessed and found eligible based on the criteria; 599 completed the self-administered questionnaire, resulting in a response rate of 98.2%.” Page 16, lines 306-307 |
|  |  | (b) Give reasons for non-participation at each stage | Yes | | Reasons for not participating included being absent on the day of data collection or refusing the invitation to participate (Page 16, lines 306-307. |
|  |  | (c) Consider use of a flow diagram | N/A | | Using a flow diagram was considered inappropriate. |
| Descriptive data | 14* | (a) Give characteristics of study participants (eg demographic, clinical, social) and information on exposures and potential confounders | Yes | | Table 1 (page 16) details the characteristics of participants, while Tables 2 (page 18) and 3 (page 19) present information on exposures and potential confounders. |
|  |  | (b) Indicate number of participants with missing data for each variable of interest | N/A | | Not applicable; no variables had missing data. |
| Outcome data | 15* | Report numbers of outcome events or summary measures | Yes | | All numbers are reported in the tables. |
| Main results | 16 | (*a*) Give unadjusted estimates and, if applicable, confounder-adjusted estimates and their precision (eg, 95% confidence interval). Make clear which confounders were adjusted for and why they were included | Yes | | Both unadjusted and adjusted estimates, along with 95% confidence intervals, are shown in Table 4. The table's footnotes explain how adjustment affects the significance of all odds ratios. |
|  |  | (*b*) Report category boundaries when continuous variables were categorized | Yes | | Category boundaries are shown in the table, where applicable (age groups, social supports, clinical cut-offs). |
|  |  | (*c*) If relevant, consider translating estimates of relative risk into absolute risk for a meaningful time period | N/A | | N/A |
| Other analyses | 17 | Report other analyses done—eg analyses of subgroups and interactions, and sensitivity analyses | Yes | | Item 17 - Addressed on page 22, lines 366-373, in the 'Results" section.  “Furthermore, Table 5 below presents the findings from testing for effect modification between gender and different types of traumas. Gender and contact sexual abuse were found to interact significantly (AOR = 2.79, 95% CI [1.85, 4.19], p < 0.001). Other interactions between trauma and gender did not show statistical significance. Stratified analyses were performed to interpret the significant interaction. The relationship between anxiety and contact sexual abuse was statistically significant for females (AOR = 4.11, 95% CI [1.31, 12.89], p = 0.015), but not for males (AOR = 2.20, 95% CI [0.38, 12.84], p = 0.381), suggesting a gender-specific effect in which females were more susceptible to anxiety after this particular trauma.” |
| Discussion | | |  | |  |
| Key results | 18 | Summarise key results with reference to study objectives | Yes | | The discussion section outlines the key results and highlights their main practical impacts on pages 27-32. They are also summarized in the conclusion. |
| Limitations | 19 | Discuss limitations of the study, taking into account sources of potential bias or imprecision. Discuss both direction and magnitude of any potential bias | Yes | | The limitations are described under the “Limitations and strengths of the study” section (page 32). |
| Interpretation | 20 | Give a cautious overall interpretation of results considering objectives, limitations, multiplicity of analyses, results from similar studies, and other relevant evidence | Yes | | References were added where possible and discussed. Limitations were addressed in the discussion. |
| Generalisability | 21 | Discuss the generalisability (external validity) of the study results | Yes | | The findings of this study are most applicable to similar school-based youth groups in post-conflict regions of the Horn of Africa. |
| Other information | | |  | |  |
| Funding | 22 | Give the source of funding and the role of the funders for the present study and, if applicable, for the original study on which the present article is based | Yes | | Funding information was clarified during submission but not included in the manuscript, as requested. |

*Give information separately for exposed and unexposed groups.

**Note:** An Explanation and Elaboration article discusses each checklist item and gives methodological background and published examples of transparent reporting. The STROBE checklist is best used in conjunction with this article (freely available on the Web sites of PLoS Medicine at http://www.plosmedicine.org/, Annals of Internal Medicine at http://www.annals.org/, and Epidemiology at http://www.epidem.com/). Information on the STROBE Initiative is available at www.strobe-statement.org.
