# Supplementary material for: Anxiety among secondary school students in the war-torn Tigray, Ethiopia, 2024: A call for action
Source: PLOS Ment Health. 2026 Mar 18;3(3):e0000526. doi: 10.1371/journal.pmen.0000526 (PMC12998859; doi:10.1371/journal.pmen.0000526)
Supplement: Attachment — Submitted filename: Response to Reviewers.docx [file pmen.0000526.s003.docx]

December 9, 2026

**Dear Editor**,

Thank you for allowing us to submit the revised draft of our manuscript entitled “Anxiety among secondary school students in the war-torn Tigray, Ethiopia, 2024: a call for action” to **PLOS Mental Health**. We appreciate the time and effort that you and the reviewers have dedicated to providing your valuable and constructive feedback on our manuscript.

We are grateful for the insightful comments and suggestions, all of which have greatly helped us to improve the clarity and quality of the paper. We have revised the manuscript accordingly and provide below a detailed, point-by-point response to all comments from the reviewers, the editor, and the editorial staff.

**Sincerely**,

Haftom Tesfay Gebremedhin, MSc.

Adigrat University, Ethiopia

E-mail: [haftomtesfay30@gmail.com](mailto:haftomtesfay30@gmail.com)

Tele: +251-969 16 44 12

I. **Point-by-point response to editors**
1. *L71 Is "controllable" the right word - most cognitive behavioral techniques are built on the assumption that thoughts are controllable and anxiety can be managed.*

**Response:** I appreciate you bringing up this crucial point. We agree and have changed "uncontrolled" to "unmanaged" (**page 5, lines 78-81**).

2. *L84 It can be inferred in this paragraph that anxiety during the conflicts themselves occurs, and chronic exposure to the stressors associated with conflict is a risk factor for later PTSD. But this isn't stated explicitly.*

**Response:** I appreciate your excellent point. The paragraph has been updated to clearly indicate the causal relationship we meant to imply (**page 5, lines 87-89**).

3. *L102 Is "higher proportion of anxiety" the right phrase, do you mean prevalence? Prevalence is not the same as developmental characteristics.*

**Response:** I appreciate your correction. To ensure terminological accuracy, we have substituted "prevalence of anxiety" for "higher proportion of anxiety" (**page 7, lines 117-118**).

4. *L115 Use "prevalence" rather than "rate" for consistency.*

**Response:** Thanks, we have substituted "prevalence" for "rate" in the manuscript (**page 5, lines 106-107**).

5. *L122 Now we have "level" in addition to "rate" and "prevalence" - be consistent throughout.*

**Response:** Thank you. We have replaced "level" with " prevalence" (**page 8, lines 139-140)**.

6. *L127 Rewrite the end of the introduction to state a clear research question, including any pre-registration and subgroup analyses (e.g. gender) proposed. Remove statements like "The findings..." which belong in the discussion (and avoid sounding journalistic). How does your question differ from the existing prevalence survey (34%) also done on youth? Not clear here.*

**Response:** We appreciate the guidance you provided. We have now revised the introduction to include a straightforward research question and to clarify how our study differs from the previous survey (**pages 7 & 8, lines 132-142**).

7. *L154 If this is not a simple random sample (you mention stratification which is not simple random sampling), you need to adjust for the complex survey design features in the analysis to obtain correct standard errors.*

**Response:** Thank you for pointing out this crucial methodological issue. Our study used a stratified simple random sampling design with proportional allocation and no clustering, ensuring equal selection probability for all students. This avoids complications from clustering and unequal weighting. Thus, we believe that standard methods like logistic regression provide reliable estimates, and we've clarified this in the revised sampling section (**page 10, lines 176–187**).

8. *L163 More detail needed on measures e.g. internal consistency reliability and validity in this country/language. Group into exposures, outcomes, covariates - justification needed.*

**Response:** We appreciate your insightful comments. To give more information about the validity and reliability of the measures and to clearly group the variables with explanations, we have updated the "Data collection and instruments" section (**pages 10-12, lines 188-229**).

9. *L200 Translation into another language does not necessarily establish reliability and validity of psychometric measures.*

**Response:** We are grateful to the editor for this critical insight. We agree that translation is not enough on its own. To establish cross-cultural validity, our revised text indicates that we employed a rigorous methodology that went beyond straightforward translation (**pages 12 & 13, lines 242-250**).

10. *L208 I think the analysis is incorrect if there was stratification, and it should be done with complex samples feature in SPSS. Otherwise the standard errors are incorrect*.

**Response:** We appreciate your important methodological point. The sampling section has been revised to clarify that a stratified simple random sample was used, ensuring every student had an equal chance of selection. Stratification was used during sampling to provide an equal chance of selection. We considered the analysis valid and the standard errors from logistic regression in SPSS to be unbiased and reliable. This explanation has been added to the manuscript (**page 10, lines 176–187**).

11. *P14 Is orthodox a religion? It is usually a subtype of a number of different religions.*

**Response:** We have revised the text accordingly, substituting “Orthodox Christianity” for “Orthodox” to correctly reflect the religious classification (**page 17, table 1**).

12. *Table 3 - the traumatic events are not specific to conflict. Are these positioned as covariates, exposures or something else? The status of your variables needs to be clear throughout.*

**Response:** We acknowledge the importance of the above comment and agree that factors related to trauma need clarification. For the current analysis, the traumatic events mentioned in Table 3, including both conflict and non-conflict traumas, were considered independent variables or exposures (**Table 3**).

13. L268 Here and elsewhere you are reporting prevalence for things not positioned as outcomes. It gives the paper an exploratory feel, lacking focus.

**Response:** Thank you for raising this issue. We have now revised the presentation of the data on suicidal ideation and attempted to eliminate the standalone reporting of prevalence (**page 19, lines 332–334**).

14. *L388 The point about persistent exposure to trauma and dysregulation of HPA axis is made well here, and needs more strongly stating in the introduction.*

**Response:** We appreciate the editor's insightful recommendation. We agree that the paper's framework is improved by bolstering the neurobiological justification in the introduction. As a result, we have included a clear explanation of how traumatic exposure can dysregulate the HPA axis and result in chronic anxiety in the introduction (**pages 5 & 6, lines 89–94**).

15. L432 Clinically significant levels of anxiety, rather than anxiety?

**Response:** Thank you for this valuable comment. We agree that precision in defining our outcome measure is crucial. In response, we have revised the manuscript to consistently use the term 'clinically significant anxiety' to accurately reflect that our findings are based on a validated clinical cut-off score from our screening instrument (**page 33, lines 523**).

II. **Point-by-point response to reviewers**

**Response to reviewer #1:**

We want to extend our honest appreciation to **Reviewer #1**, whose comments were thoughtful and constructive. We are grateful for the time and expertise contributed to the review of our manuscript and have addressed all comments and suggestions in this revised version. Please find below our responses to each point raised.

1. *Critical Gap: The manuscript lacks a clear theoretical framework to guide the understanding of post-conflict anxiety development. While the authors cite relevant literature, they fail to articulate how war trauma specifically manifests in anxiety disorders among adolescents or what theoretical model underlies their variable selection.*

**Response:** We appreciate the reviewer’s constructive feedback and agree on the importance of a strong theoretical basis. We have incorporated an Integrated Ecological-Transactional Framework, based on Bronfenbrenner's Ecological Systems Theory and Lazarus and Folkman’s Transactional Model of Stress and Coping. Significant revisions have been made, detailed on **page 7, lines 124-131**.

2. *The study is limited to one town (Adigrat), significantly limiting generalizability to the broader Tigray region or other post-conflict contexts.*

**Response:** We appreciate the reviewer’s important observation regarding the generalizability of our findings. While the issue affected the entire region, we recognize that our focus on a single town is a limitation of our study. We have explicitly acknowledged this limitation in the revised "Limitations" section of the manuscript (**page 32, lines 512-514**).

3. *The exclusion criteria mention 'seriously sick' students but provide no operational definition.*

**Response:** We really appreciate the reviewer pointing this out. We totally agree that having a clear, operational definition is essential. So, we’ve gone ahead and updated the Methods section. Instead of using the term "seriously sick," we’ve opted for a more precise definition that focuses on how well individuals can participate. You can find the changes on **page 9, Lines 163-168**.

4. *No discussion of potential selection bias through school-based sampling (excluding out-of-school youth who may have higher trauma exposure).*

**Response:** We appreciate the reviewer's insightful comment. We agree that the school-based sampling frame is a drawback since it leaves out young people who are not enrolled in school, who might be more vulnerable to trauma exposure and its aftereffects. As a result, we revised the manuscript to include it as a study limitation in the Limitations section (**page 32, lines 514-517**).

5. *The inability to establish temporal relationships between trauma exposure and anxiety symptoms is particularly problematic when studying post-conflict populations.*

**Response:** We thank the reviewer for this crucial comment regarding the limitations of cross-sectional design. We fully agree and have addressed this concern in the Limitations section of the manuscript (**page 32, lines 509-511**).

6. *No baseline pre-conflict data for comparison, despite referencing a wartime study with similar prevalence rates.*

**Response:** We appreciate the reviewer's important observation and feedback. We used the pre-conflict anxiety prevalence of 11.48% from the study conducted in Tigray as a contextual baseline, and we have revised the introduction and discussion sections accordingly (**page 6, lines 105-106**).

7. While the authors mention translation procedures, there is insufficient discussion of the cultural validity of Western-developed instruments (GAD-7, PHQ-9) in the Ethiopian context.

**Response:** We appreciate the reviewer for bringing up this important point. In response, we have clarified in the Methods section that our process included not just linguistic translation but also formal checks for conceptual and cultural equivalence.

8. *The dichotomization of continuous anxiety scores (GAD-7 ≥10) may result in loss of important information about anxiety severity gradations*

**Response:** We appreciate the reviewer’s insightful comment on the dichotomization of anxiety scores and its effects on statistical power. We selected the standard cut-off of ≥10 from previous literature to align with clinical practice for identifying "probable cases" of anxiety disorders. However, we acknowledge the importance of considering the full spectrum of severity.

9. *Several trauma variables appear to have small cell sizes that could affect model stability*

**Response:** We appreciate you pointing out that some trauma variables have small cell sizes. We are aware of this restriction. We thoroughly examined problems associated with model instability (such as inflated standard errors and wide confidence intervals), even though we did not collapse categories or eliminate these variables. The overall model fit was satisfactory, and the estimates stayed consistent.

10. *No exploration of interaction effects between key variables (e.g., gender × trauma exposure)*

**Response:** We appreciate your comment. The interaction effects between gender and the different types of trauma exposures were investigated. These findings have been included and discussed, as can be seen in **Table 5** and the revised manuscript (**page 22, lines 366-373**).

11. *Limited discussion of effect sizes and clinical significance of associations*

**Response:** We appreciate the reviewer's thoughtful observation. To interpret the strength of the observed associations and their clinical/public health significance, we have expanded the discussion section.

12. *No sensitivity analyses to test robustness of findings*

**Response:** Thank you for your comment. To conduct a sensitivity analysis, we fitted several multivariable logistic regression models that included only the variables significant in the bivariate analysis at p < 0.05. The results confirmed the robustness of our findings, as they aligned with those of the main model. We have now updated the data analysis section to reflect this (**page 14, lines 267-270**).

13. W*riting Quality: The manuscript contains numerous grammatical errors and awkward constructions that impede readability. Professional editing is recommended.*

**Response:** We thank the reviewer for this valuable feedback. In response to this comment, we have carefully edited the manuscript to fix all grammatical errors, improve sentence flow, and make it more readable.

14. *Table Formatting: Tables 4 particularly would benefit from better formatting to improve interpretability of results.*

**Response:** We appreciate the reviewer for this useful suggestion. We have reformatted **Table 4** to improve its clarity and make the results easier to interpret.

15. *Literature Integration: The discussion appropriately contextualizes findings within existing research but could benefit from deeper engagement with post-conflict mental health literature from sub-Saharan Africa.*

**Response:** Thank you for your feedback on our literature integration. We appreciate your suggestion to engage more with post-conflict mental health literature from sub-Saharan Africa. We understand how important this work is for providing context to our findings, and we tried to strengthen the discussion accordingly.

16. *Limitations Section: While present, the limitations discussion understates several key methodological concerns, particularly regarding generalizability and causal inference.*

**Response:** Thank you for your feedback. We have added discussions on generalizability and causal inference in the limitations section (**page 32, lines 509-517**).

17. *Line 150-153: The sample size calculation should specify the actual proportion used (39.7%) more clearly in the methods rather than just referencing another study.*

**Response:** We appreciate the reviewer's suggestion. We have updated the methods section to present the proportion more clearly (**page 9, 171-174**).

18. *Line 277-281: The gender-specific prevalence rates (47.4% for females, 12.9% for males) are consistent with the overall rate and gender distribution in the sample.*

**Response:** We appreciate the reviewer's comment. They are correct that the gender-specific prevalence rates align with the overall prevalence and gender distribution in our sample. This is addressed in the revised manuscript on **page 21, lines 343-345**. As the reviewer pointed out, the overall prevalence of 32.7% results from the higher rate of anxiety among females (163 out of 344 equals 47.4%) and the fact that females made up a larger portion (57.4%) of the sample than males (42.6%). We have also used this opportunity to verify that all confidence intervals reported in this section are correct.

19. *Lines 305-314: The comparison with wartime prevalence is intriguing but needs more careful interpretation given different methodologies and timing.*

**Response:** We appreciate the reviewer for raising this critical point. We agree that different methods and contexts make it hard to compare numbers directly. We have updated the discussion to soften our interpretation and present the similarity in prevalence as a suggestive and concerning pattern rather than as direct equivalence (**page 27, lines 390-391**).

20. *Lines 432-437: The conclusion appropriately calls for targeted interventions but could benefit from more specific, actionable recommendations based on the study's risk factor findings.*

**Response:** We appreciate the reviewer's helpful feedback. We have revised the conclusion to offer clear, practical recommendations based on the main risk factors identified in our study (**page 33, lines 527-532**).

21. *There is an inconsistency in the manuscript regarding contact sexual abuse odds ratios - the abstract reports AOR=3.09 (95% CI: 1.21, 7.91) while Table 4 and the results text show AOR=3.97 (95% CI: 1.58, 9.96). This discrepancy requires correction.*

**Response:** We thank the reviewer for their careful attention to detail in spotting this discrepancy. The value in the abstract OR was a typographical error and has been corrected to match the correct result shown in Table 4 and the results section (**page 3, line 54**).

22. *Inconsistent citation formatting*

**Response:** We thank the reviewer for highlighting this issue. We carefully reviewed the entire manuscript and made sure all in-text citations and reference list entries follow the Plos Mental Health's formatting guidelines consistently. We used reference management software (EndNote) to ensure the correct style was applied throughout.

23. *Figure legends could be more descriptive*

**Response:** We appreciate the reviewer's helpful suggestion. We have updated all figure legends to make them more detailed and more precise.

24. *Some statistical reporting lacks precision (confidence intervals, exact p-values)*

**Response:** We thank the reviewer for this valuable feedback. We have thoroughly revised the manuscript. Now, all statistical results report exact p-values and include confidence intervals where necessary.

**Response to reviewer #2:**

We genuinely appreciate Reviewer #2's insightful and helpful comments and suggestions on our manuscript. We appreciate your acknowledgment of the significance of our work and thank you for the insightful feedback that has improved the manuscript's overall quality and clarity. We address each issue raised in detail and point-by-point below.

1. *There is not enough data regarding the questionnaires used (aside of the GAD-7). Details have to be added*

**Response:** We appreciate the reviewer’s feedback. We have expanded the methods section to include detailed descriptions of all the questionnaires used in the study. We believe the manuscript now offers enough detail on all the instruments (**pages 10-12, lines 191-230**).

2. *Why was the score 10 on the GAD-7 used as the cut-off for "anxiety"? This needs to be explained given the centrality of this variable in the manuscript. Same goes for the cut-off score for "depression", though it is less critical.*

**Response:** We appreciate the reviewer's essential question. We have now explained the rationale for using the cut-off in the manuscript (**pages 10 & 11, lines 193-206**).

3. *I was surprised to read that the ages of the students in the study sample were up to 24. In many parts of the world individuals graduate high school at the ages of 17-19. This needs to be briefly addressed.*

**Response:** We appreciate the reviewer's insightful observation. The broader age range in our sample, which includes students up to 24 years old, directly reflects the major disruptions to the education system caused by the war in the region.

4. *In relation to the comparison of the study results regarding prevalence of anxiety to previous studies I am doubtful that the difference between studies of few percent is meaningful. I particularly point here to what is presented in lines 326-327.*

**Response:** We thank the reviewer for highlighting this critical point. In our manuscript, we have already used the confidence interval around our point prevalence (32.7%, 95% CI: 28.9%, 36.5%) as the basis for comparison. This helps us avoid overinterpreting minor differences in point estimates (**page 27, lines 384-386**).

5. *Moreover, the segment of the discussion dealing with this comparison could be much more concise. And more generally, the discussion section as a whole is rather lengthy.*

**Response:** We appreciate the reviewer's helpful suggestion to make the discussion more concise and impactful. In response, we have revised the entire discussion section.

6. *Though the manuscript is overall easily readable, there are some problems with grammar and overall quality of writing. The authors should have a professional overgoing their manuscript in order to correct grammar mistakes, use correct terms and avoid unnecessary repetitions and redundant sentences (such as the one in lines 254-255).*

**Response:** We appreciate the reviewer's feedback regarding the redundant phrasing in lines 254-255. We agree that the sentence about the study involving 599 secondary students is unnecessary, as the context is clear. We have also made grammatical corrections and improved the manuscript's quality of writing (**page 17, line 318**).

**Response to Reviewer #3:**

We would like to sincerely thank Reviewer #3 for carefully reviewing our manuscript and for the helpful comments he offered. Your suggestions and insights have been extremely helpful in enhancing the overall impact of our study. We have given each point careful thought, and our thorough responses are provided below.

1. *Too generic: The background starts with a general statement:*

*"Anxiety is a common mental health issue that significantly affects the daily life of human beings." – This is overly broad and somewhat generic. It doesn't yet set up a compelling case for why anxiety needs to be studied in this specific population and setting.*

*The transition to "particularly in conflict zones" is useful, but the connection between war, anxiety, and post-conflict settings needs much stronger development.*

*Lack of specificity: It mentions "conflict zones" and "post-conflict areas" without naming the specific context (Tigray) until the last line.*

*Missing significance: It doesn't tell us why this study is important — for public health, policy, or local recovery efforts.*

*Weak transition to objective: The jump from "limited evidence" to "this study aims..." is too abrupt and unsubstantiated.*

**Response:** We appreciate the reviewer's insightful comments on the need for a more transparent, more compelling background. We have completely rewritten the background to address these points directly. **Lines 35–42 on page 3** of the updated manuscript contain the revisions.

Specifically:

- To improve the opening and add specificity, we now start by clearly defining the context of the war in Tigray and its impact on adolescent mental health. This replaces the general statement about anxiety.
- To strengthen the link between war and anxiety, we discuss how psychological trauma lasts in post-conflict settings, especially for adolescents.
- To clarify the significance, we explain why this study is essential.
- To create a seamless transition, we now present a straightforward narrative from the identified research gap to our study’s goal, ensuring it is well-supported.

2. *The results are clearly presented. It would be much better if you could do grouping or prioritizing the most impactful predictors. Because. It feels slightly overwhelming to list 8 risk factors one after another — can feel like a data dump.*

**Response:** We appreciate your insightful comment. We acknowledge that listing numerous predictors one after the other may seem overwhelming. As a result, we have rearranged the presentation of results by classifying the predictors into thematic categories (sociodemographic factors, exposures related to conflict and violence, and factors related to mental health). We think that this reorganization enhances the interpretability and clarity of the results (**pages 3 & 4, lines 48-56**).

3. *The conclusion: Vague: "high prevalence” compared to what? Other studies? National estimates? Could say how the findings can inform interventions (e.g., school-based screening, trauma services).*

**Response:** Thank you for your helpful feedback. We agree that the phrase “high prevalence” was vague and insufficiently contextualized for the findings. We have rewritten the conclusion to specify the level of anxiety observed in our study and explain how it compares to previous evidence. We also improved the implications section to show better how the findings can support school-based screening, trauma-focused services, and mental health programs for adolescents and youth after war. These changes enhance the clarity and practical importance of the conclusion (**page 4, lines 57-61**).

4. *I suggest reconsidering how the introduction opens. While defining anxiety has its place, starting with a textbook-style definition may not immediately engage the reader or highlight the significance of your study. Given your focus on a post-conflict region, it would be more impactful to begin by framing the broader issue — that war-affected populations, particularly adolescents, are at heightened risk of anxiety and other mental health problems. This would help set the stage for your study in Tigray and guide the reader more clearly toward your research objective.*

*Overall, the introduction contains a considerable amount of general information on anxiety — including definitions, global statistics, and clinical descriptions — but it takes too long to get to the specific problem this study aims to address.*

**Response:** We agree with this great suggestion. The revised introduction was organized using a "funnel" approach (**pages 5–8, lines 72–143**). It started by highlighting the critical public health issue of mental health in adolescents affected by war. Then we narrowed the focus to the specific post-conflict situation in Tigray, Ethiopia. This leads directly to a clear statement of the research gap and our study's objective.

5. *It would strengthen the justification for the study to clarify a few important points. First, since anxiety and depression frequently co-occur with PTSD in post-conflict settings, it would be helpful to explain how your study specifically addresses anxiety as distinct from PTSD, or whether comorbidity was considered in your assessment.*

**Response:** We appreciate you bringing up this important point. We revised the introduction to clearly explain why anxiety deserves a targeted study that is separate from, but frequently co-occurs with, depression and PTSD (**pages 5–8, lines 72–143**). We included text that clarifies that anxiety disorders are a distinct group of conditions marked by persistent worry and apprehension about the future, which is highly pertinent to teenagers in a region that is recovering. Additionally, our methodology stated that our assessment tool (GAD-7) was explicitly selected to capture anxiety symptoms (**pages 10 & 11, lines 193-206**).

6. *the paper mentions that anxiety prevalence has reportedly tripled in the post-conflict period (to around 34%), but it would be important to clarify what the baseline was and how this compares not just globally, but also within other regions of Ethiopia. For instance, how does the prevalence of anxiety in Tigray compare with non-conflict regions of Ethiopia? What makes Tigray’s situation uniquely deserving of focused study? While some relevant comparisons and previous studies are mentioned in the discussion section, it would be more appropriate to bring these into the introduction. Doing so would provide readers with a more complete picture of the existing evidence — both globally and locally — and more clearly articulate the research gap your study is addressing.*

**Response:** Thank you for raising this helpful recommendation to improve the context. We incorporated comparative data into the introduction. To provide a clear baseline and contrast, this entails citing specific prevalence rates from international studies as well as, crucially, from other Ethiopian regions (including non-conflict areas). The intensity of the conflict, the systematic breakdown of institutions, and the timing of our study in the immediate, precarious post-agreement period are the factors that make Tigray's situation particularly severe.

7. *While the traumatic exposures listed in the introduction (e.g., displacement, violence, health system collapse) certainly highlight the severity of the conflict, listing these alone doesn’t fully justify an anxiety-specific study. These exposures are commonly cited in research on PTSD or depression, and the way they're presented here seems to support a broader mental health burden rather than a focused anxiety investigation. To better justify the study, I suggest explaining how these specific types of trauma are known to contribute to heightened anxiety — especially in adolescents — and why this deserves focused attention, independent of PTSD or depression*

**Response:** We appreciate your thoughtful comment. We agree that an anxiety-focused study could not be justified by merely listing traumatic exposures. As a result, we have significantly improved the introduction by elucidating how the conflict-related stressors encountered in Tigray lead to increased anxiety in youths.

First, we added evidence of how trauma and long-term stress affect neurobiological systems, specifically the amygdala and the hypothalamic-pituitary-adrenal (HPA) axis, which increases susceptibility to chronic anxiety symptoms, to biological and developmental justification. Second, we made it clear that anxiety is not only an immediate response to conflict but can also get worse over time, as shown by long-term research on young people affected by war. Third, we compared Ethiopia's anxiety levels before and during the war to highlight the dramatic increase in symptoms and the importance of researching anxiety in this setting.

To explain how ecological disruption, trauma exposure, and personal coping mechanisms interact to shape adolescent anxiety trajectories in post-conflict settings, we finally improved the theoretical foundation by more clearly integrating Bronfenbrenner's Ecological Systems Theory and the Transactional Model of Stress and Coping.

These revisions directly offer a convincing argument for emphasizing anxiety as an independent outcome (**pages 5–7, lines 85–132**).

8. *Additionally, the manuscript mentions multiple times that the study is “unique,” but this needs to be clarified. If the uniqueness lies in the timing (post–Pretoria Peace Agreement), the population (secondary school students), or the focus on anxiety as a primary outcome, that should be explicitly stated and justified.*

**Response:** We appreciate your feedback. We have now made it clear that the novelty of our work depends on three different factors: first, its crucial timing as one of the first mental health surveys carried out after the Pretoria Peace Agreement; second, its particular focus on adolescents in a school setting, an important but understudied group; and third, its primary focus on anxiety, a condition that is frequently overlooked in post-conflict research due to PTSD and depression (**pages 7 & 8, lines 133-143**).

9. *This section includes some context that, while important in general, may not be directly relevant to the study's design, setting, and timeframe. If I correctly understand what you were saying, for example, mentioning the decrease in the number of functioning schools — possibly due to conflict-related damage — feels more like a journalistic narrative than a methodological description.*

**Response:** Thank you for your thoughtful comment. We recognize that some of the information in the original "Design, setting, and period" section seemed like a narrative and didn't relate directly to the methods we described. We aimed to include the total number of primary and secondary schools in 2013 to provide context for the overall number of schools in the town. We wanted to clarify that only five (four public and one private) are secondary schools.

In response, we have updated the section to focus only on the current number of secondary schools and the present student population, as these details are directly relevant to the study of design and sampling (**page 9, lines 157-159**).

10. *As a researcher, it’s important to maintain focus on the core components of your study design. I understand and sympathize with the broader challenges the region faces, but in this section, I would recommend keeping the reader’s attention on the information essential to understanding where, when, and how the study was conducted. Consider moving context about the state of the “education system” (usually system a nationwide-program in my understanding) to a background or discussion section if you feel it’s necessary to include.*

**Response:** Thank you for the helpful comment. We agree that the background information about the broad education system was not necessary for the “Design, setting, and period” section. Therefore, we have removed this information to keep the focus on the methodological details about where, when, and how the study took place (**page 9, lines 158-160**).

11. *I believe the numerical data of the population based on gender, if possible, including the school and grade information belongs here.*

**Response:** We appreciate your recommendation. We agreed and revised the 'Study Participants' section to comprise the numerical data according to school type (public vs. private) and gender (**page 9, lines 161-163**).

12. *For better presentation, the inclusion and exclusion criteria need to be further elaborated with reasons to justify your decision.*

**Response:** We appreciate your suggestion. The 'Study Participants' section has been updated with a more thorough explanation of the inclusion and exclusion criteria (**page 9, lines 164-169**).

13. *The description of participants as a "single population" could be misleading, as they appear to differ significantly across several dimensions — including gender, school type (private vs. public), and war-related experiences (combatants, victims…) because they were your important predictors. These subgroups may have very different exposure profiles or risk factors for anxiety. It would be helpful to clarify what is meant by "single population" in this context. If this refers only to the target group being "secondary school students in Adigrat," that should be clearly stated to avoid confusion.*

**Response:** We appreciate your thoughtful observation. We agreed that the term "single population" may have been confusing when used in the sample size determination. To be clear, the term refers only to the target population, which is uniformly defined as "all secondary school students in Adigrat Town," from which the overall sample was drawn. To make this point clear and prevent any misunderstandings, we revised the manuscript (**page 9, lines 171-172**).

14. *It’s not fully clear how randomization was implemented at the stratum level. While the use of a computer-generated lottery is mentioned, it would help to explain how student identification numbers were obtained for each grade and school type (public/private), and how feasible it was to access and use this full list in practice. Was a complete list available for each stratum, or were students asked to volunteer and then selected randomly? Providing a bit more detail here would clarify the sampling procedure and reassure readers about the randomness of participant selection.*

**Response:** We appreciate your asking for this crucial clarification. The 'Sampling Technique' section has been revised to give a more precise and more thorough explanation of the randomization procedure (**page 10, lines 177-189**).

15. In the instruments section, I recommend giving equal attention to the description of all tools used in the study. Currently, some instruments are briefly mentioned, while others (like the General Anxiety Disorder-7 (GAD-7) were described in more detail. It would improve clarity and consistency to follow a similar structure for each instrument — including source, number of items, scoring, and whether it was self- or interviewer-administered.

**Response:** We thank the reviewer for this valuable observation. We agree that there were inconsistencies in the description of the data collection tools. We revised the "Instruments" section to include a uniform, equally thorough description of every instrument used in our study (**pages 10-12, lines 190-230**).

16. *Additionally, the statement that suicidal ideation and attempts were assessed using questions adopted from the WMH-CIDI might be confusing. As far as I understand, the WMH-CIDI is a structured diagnostic interview typically administered by trained professionals, not self-administered. If you only used selected items from the CIDI within a self-administered questionnaire, it would be important to clarify that, so as not to give the impression that formal clinical diagnoses were made.*

**Response:** We appreciate this crucial explanation. We certify that the word "adopted" was misused. The correct word to use is "adapted."

We revised the manuscript to correctly indicate that the suicidality questions were modified from the WMH-CIDI and transformed into straightforward, self-administered "Yes/No" items for this survey (**page 11, lines 217-219**).

17. *While the reported Cronbach's alpha of 0.97 for the GAD-7 indicates excellent internal consistency, such a high value may also suggest that some items are overly similar or redundant. This can sometimes occur when forward or backward translations result in items sounding too alike — particularly in languages with limited emotional vocabulary. It would be helpful to include a brief note in the discussion section addressing this possibility, to reassure readers that the high internal consistency does not compromise the scale’s validity or interpretability in your context.*

**Response:** We appreciate the reviewer's insightful observation. The correct value determined from our data is 0.917; the reported Cronbach's alpha of 0.97 was a typo. We recognize that high value may raise concerns about possible item redundancy, which may have been affected by the translation process (**page 11, lines 205-206**).

18. *The current analysis approach is generally appropriate, but there are areas that could be improved. Relying on p-values from bivariate analysis to select variables for the multivariable model may overlook important confounders. It would be better to select variables based on theoretical relevance or a conceptual framework.*

**Response:** We appreciate your insightful observation of our variable selection procedure. You are right to point out that a purely statistical approach may miss significant confounders. To identify potential variables and incorporate those with a priori theoretical relevance to our conceptual framework, we used a bivariate association criterion with p ≤ 0.25 as an initial screening criterion. A bivariate association of p ≤ 0.25 was the criterion for variable inclusion, not p ≤ 0.05, as correctly stated on **page 13, lines 262–264**. We apologize for the typographical error that resulted in the stated p-value of ≤ 0.05.

19. *Additionally, reporting more diagnostic measures (e.g., pseudo R², AUC) and considering interaction terms could strengthen the model.*

**Response:** As recommended, we have now included more diagnostic measures to offer a more thorough evaluation of our model. The revised manuscript has an Area Under the Curve (AUC) of 0.905 (95% CI [0.88, 0.930], p <.001), indicating exceptional predictive accuracy, and a Nagelkerke R² value of 0.578, indicating significant explanatory power (**page 14, lines 273–275**).

At your suggestion, we conducted interaction analyses of gender and trauma types. This showed that the interaction of contact sexual abuse and gender was significant (AOR = 2.79, 95% CI [1.85, 4.19], p < 001), and we have now probed, analyzed, and discussed it (**page 14, lines 277–281**).

20. *If anxiety severity levels were used, ordinal logistic regression might offer more nuanced insight.*

**Response:** We appreciate the reviewer's suggestion. We aimed to determine factors associated with the presence or absence of clinically significant anxiety (a binary outcome), given its potential impact on public health screening and intervention in this setting. We recognize that assessments of severity levels are an essential direction for future, more refined investigation.

21. *Finally, clarification on how missing data were handled would improve the transparency of the analysis.*

**Response:** We are grateful for the reviewer's feedback on the missing data. The final dataset had no missing values; no imputation was required because built-in validation and mandatory field checks during EpiData entry prevented missing values.

22. *The reported anxiety prevalence (32.7%) is clearly concerning and deserves attention, but I would caution against characterizing it as exceptionally high without more context. The manuscript frequently refers to a “threefold increase” in anxiety during the war (34%) compared to pre-war levels, but no data or references are provided to support this claim — neither for pre-war Tigray nor for Ethiopia more broadly.*

**Response:** We appreciate your insightful comment. You're right that our initial manuscript made a bold assertion without offering the relevant comparison information. To appropriately contextualize our findings and support the reported increase, we have now updated the text to include precise, citable pre-war and during-conflict prevalence rates from the pertinent literature (**page 27, lines 386-395**).

23. *This same comparative statement appears across several sections (introduction, methods, results, and now discussion), and yet no baseline data is cited.*

**Response:** We appreciate your insightful comment. You are right that our initial manuscript made repeated references to a comparative change without offering supporting information. In the updated manuscript, we have now fully addressed this problem.

To support the claim, we have compiled a discussion of this trend and, above all, introduced precise baseline and wartime prevalence data. To frame the significance of the study, the pre-war prevalence for Tigray (11.48%) and the during-conflict prevalence (34.43%) from the cited phone survey are now stated explicitly in the introduction and again in the discussion to explain our findings (**page 6, lines 106-108**).

24. *If you are aware of existing studies or national surveys that report pre-conflict anxiety rates in Ethiopia, those should be included in the background section to help readers understand the magnitude and significance of your findings. If no such data exists, it would be better to either acknowledge the lack of baseline data or reframe the comparison more cautiously.*

**Response:** We appreciate your insightful recommendation. We agree that baseline data is essential for framing the significance of our findings. In response, we updated the introduction's background section with precise, citable prevalence estimates of pre-conflict anxiety (**page 6, lines 103-109**).

25. *In the current form, this central comparison risks overstating the case and may mislead readers about the actual mental health trajectory before, during, and after the conflict*.

**Response:** We appreciate this critical insight. We agree that the comparison could be misleading without precise data. We have completely revised our presentation to provide an accurate, evidence-based mental health trajectory in response to this feedback (**page 27, lines 387-394**).

26. *Putting findings like in the Woldia Town study might give background if you put it in the introduction setting.*

**Response:** We appreciate your suggestion. The prevalence from the Woldia Town study has been added to the introduction to justify our study's focus better and provide crucial context for anticipated anxiety levels in conflict-affected areas (**page 6, lines 101-102**).

**Response to Reviewer #4:**

We acknowledge the editorial note about Reviewer 4's report. Following the editor's instructions, we have ignored this report and made no changes based on it.
