# Supplementary material for: Anxiety among secondary school students in the war-torn Tigray, Ethiopia, 2024: A call for action
Source: PLOS Ment Health. 2026 Mar 18;3(3):e0000526. doi: 10.1371/journal.pmen.0000526 (PMC12998859; doi:10.1371/journal.pmen.0000526)
Supplement: Attachment — Submitted filename: Response to Reviewer_2.docx [file pmen.0000526.s004.docx]

February 15, 2026

**Dear Editor,**

We are pleased to resubmit our revised version of our manuscript, entitled “Anxiety among secondary school students in the war-torn Tigray, Ethiopia, 2024: a call for action,” to **PLOS Mental Health**. We want to express our sincere gratitude to you and the reviewers for the valuable time and effort invested in providing constructive comments on our manuscript.

We appreciate the thoughtful comments and suggestions received, as they have significantly contributed to enhancing the quality of our manuscript. We have carefully considered all the comments received and have made the relevant changes to our manuscript. In the sections below, we have provided a detailed point-by-point response to all the comments received from the reviewers, editor, and editorial staff.

**Sincerely**,

Haftom Tesfay Gebremedhin, MSc.

Adigrat University, Ethiopia

E-mail: [haftomtesfay30@gmail.com](mailto:haftomtesfay30@gmail.com)

Tele: +251-969 16 44 12

I. **Point-by-point response to editors**
1. **Comment:** *Figure Resubmissions:*

*While revising your submission, we strongly recommend that you use PLOS’s NAAS tool (*[*https://ngplosjournals.pagemajik.ai/artanalysis*](https://ngplosjournals.pagemajik.ai/artanalysis)*) to test your figure files. NAAS can convert your figure files to the TIFF file type and meet basic requirements (such as print size, resolution), or provide you with a report on issues that do not meet our requirements and that NAAS cannot fix.*

*After uploading your figures to PLOS’s NAAS tool -* [*https://ngplosjournals.pagemajik.ai/artanalysis*](https://ngplosjournals.pagemajik.ai/artanalysis)*, NAAS will process the files provided and display the results in the "Uploaded Files" section of the page as the processing is complete. If the uploaded figures meet our requirements (or NAAS is able to fix the files to meet our requirements), the figure will be marked as "fixed" above. If NAAS is unable to fix the files, a red "failed" label will appear above. When NAAS has confirmed that the figure files meet our requirements, please download the file via the download option, and include these NAAS processed figure files when submitting your revised manuscript.*

**Response:** We thank the editorial office for this guidance. All figure files have been uploaded to the PLOS NAAS tool. The processed files that meet the journal’s technical requirements have been downloaded and included in the revised submission.

II. **Point-by-point response to reviewers**

**Response to reviewer #2:**

We wish to extend our sincere thanks to **Reviewer #2** for the thoughtful and constructive comments. We appreciate the time and expertise devoted to the review of our manuscript. All the comments and suggestions have been incorporated into this revised version. Please find below our responses to all the points.

1. **Comment:** *Data collection and instruments: The description of the questionnaires is now more detailed, which is an improvement. Nevertheless, I take issue with the use of the term “anxiety” to describe participants who scored 10 or higher out of 21 on the GAD-7. Anxiety exists on a continuum, and using this terminology is imprecise. The authors should adopt more accurate language, such as “moderate to severe anxiety.” The same concern applies to the terminology used for “depression,”.*

**Response:** Thank you for your important comment. We agree that the GAD-7 and PHQ-9 tools measure the severity of the symptoms and do not offer a clinical diagnosis. We have thus changed the wording of the manuscript to reflect “moderate to severe anxiety symptoms” and “moderate to severe depressive symptoms” instead of “anxiety” and “depression.”

2. **Comment:** *Age of participants: In response to my previous comment regarding the age range of the participants, the authors state that “the broader age range in our sample, which includes students up to 24 years old, directly reflects the major disruptions to the education system caused by the war in the region.” This explanation is reasonable, but it must be explicitly addressed and discussed in the manuscript itself, as it has important implications for comparisons with high school student samples in most other countries.*

**Response:** We understand this point. We agree that the broader range impacts the manner in which we can compare this range with secondary school studies from other countries. We have therefore revised the manuscript to address this point. We explained this point in the Methods section to indicate that the range was due to educational disruption because of the war in the region (**page 9, lines 165-167**). We also discussed this point in the Limitations section (**page 30, lines 476-477**).

3. **Comment:** *Discussion section: I remain unconvinced that the differences between the anxiety rates reported in the current study and those reported in previous studies (e.g., 26.6% in Uganda or 39.7% in Ethiopia) are sufficiently large to warrant an extensive and detailed discussion. In fact, it would have been more surprising had the rates been very similar. It should suffice to note that anxiety rates differ across war-affected countries, with some variation likely attributable to differences in methodology, characteristics of the conflicts, and contextual factors. This point could be adequately addressed in a single, concise paragraph.
   More generally, the revised Discussion section remains overly long. For example, there is no need to elaborate on the correlation between anxiety and depression, which is a well-established finding in the literature.*

**Response:** We appreciate this thoughtful comment. We agree that the differences in anxiety prevalence across war-affected areas do not require extensive interpretation. Therefore, we have condensed this section into a single, clear paragraph. We noted that differences across studies may reflect methodological and contextual factors. Furthermore, we have significantly shortened the content in the Discussion section as a whole. Also, we have eliminated the extensive discussion regarding the correlation between anxiety and depression. Instead, we have briefly acknowledged that this observation was in line with previous literature.
