# Supplementary material for: Anxiety among secondary school students in the war-torn Tigray, Ethiopia, 2024: A call for action
Source: PLOS Ment Health. 2026 Mar 18;3(3):e0000526. doi: 10.1371/journal.pmen.0000526 (PMC12998859; doi:10.1371/journal.pmen.0000526)
Supplement: Attachment — Submitted filename: PMEN-D-25-00280R2_Response to Reviewer.docx [file pmen.0000526.s005.docx]

February 26, 2026

**Manuscript ID:** PMEN-D-25-00280R2

**Title:** Anxiety among secondary school students in the war-torn Tigray, Ethiopia, 2024: a call for action

Dear Editor,

We thank you and the reviewer for the careful evaluation of our manuscript and for the positive and constructive feedback throughout the review process. We sincerely appreciate the time and effort spent reviewing our work and the opportunity to revise the manuscript.

We are pleased to submit the revised version of our manuscript and have addressed the editor’s comment, as outlined below in the point-by-point response. We hope the revised manuscript meets the journal’s expectations, and we thank you for your consideration.

Sincerely,

Haftom Tesfay Gebremedhin, MSc.

Adigrat University, Ethiopia

**E-mail:** haftomtesfay30@gmail.com

**Tel:** +251-969 16 44 12

**Point-by-point response to editors**

**Comment:** *Methods. Sample size and sampling technique: “a total of 608 samples were used in this study” – should it possibly be “the total sample of 608 students was used in this study”, or “the total sample size for this study was 608”?*

**Response:** Thank you for this helpful suggestion. We agree that the wording requires clarification. The sentence has been revised accordingly. This revision is included in the Methods section under “Sample size and sampling technique” in both the tracked changes and clean versions of the manuscript.
